# Thermodynamic Stability of Mn(II) Complexes with Aminocarboxylate Ligands Analyzed Using Structural Descriptors

**DOI:** 10.1021/acs.inorgchem.2c02364

**Published:** 2022-08-22

**Authors:** Rocío Uzal-Varela, Francisco Pérez-Fernández, Laura Valencia, Aurora Rodríguez-Rodríguez, Carlos Platas-Iglesias, Peter Caravan, David Esteban-Gómez

**Affiliations:** † Centro de Investigacións Científicas Avanzadas (CICA) and Departamento de Química, Facultade de Ciencias, Universidade da Coruña, 15071 A Coruña, Galicia, Spain; ‡ Departamento de Química Inorgánica, Facultad de Ciencias, Universidade de Vigo, As Lagoas, Marcosende, 36310 Pontevedra, Spain; § The Institute for Innovation in Imaging and the A. A. Martinos Center for Biomedical Imaging, Massachusetts General Hospital, Harvard Medical School, 149, 13th Street, Suite 2301, Charlestown, Massachusetts 02129, United States

## Abstract

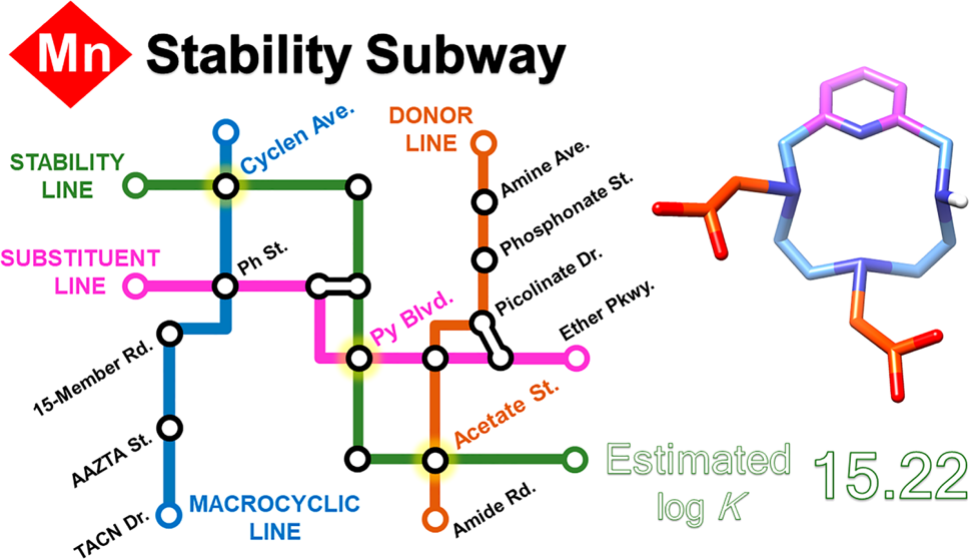

We present a quantitative analysis of the thermodynamic stabilities of Mn(II) complexes, defined by the equilibrium constants (log *K*_MnL_ values) and the values of pMn obtained as −log[Mn]_free_ for total metal and ligand concentrations of 1 and 10 μM, respectively. We used structural descriptors to analyze the contributions to complex stability of different structural motifs in a quantitative way. The experimental log *K*_MnL_ and pMn values can be predicted to a good accuracy by adding the contributions of the different motifs present in the ligand structure. This allowed for the identification of features that provide larger contributions to complex stability, which will be very helpful for the design of efficient chelators for Mn(II) complexation. This issue is particularly important to develop Mn(II) complexes for medical applications, for instance, as magnetic resonance imaging (MRI) contrast agents. The analysis performed here also indicates that coordination number eight is more common for Mn(II) than is generally assumed, with the highest log *K*_MnL_ values generally observed for hepta- and octadentate ligands. The X-ray crystal structure of [Mn_2_(DOTA)(H_2_O)_2_], in which eight-coordinate [Mn(DOTA)]^2–^ units are bridged by six-coordinate exocyclic Mn(II) ions, is also reported.

## Abstract 2

We present empirical relationships that allow estimating the log *K* and pMn values of Mn(II) complexes relevant as contrast agents for magnetic resonance imaging (MRI). The prediction of complex stability with these expressions relies on structural descriptors, providing a very powerful tool to aid with ligand design.

## Introduction

Magnetic resonance imaging (MRI) often uses contrast-enhanced procedures to attain a more accurate diagnosis of different malignancies.^1−4^ The contrast agents (CAs) that are currently used in clinics are complexes with the paramagnetic metal ion Gd(III),^5,6^ which are very efficient relaxation agents of water proton nuclei in their vicinity. As a result, CAs shorten significantly the water longitudinal relaxation times, providing an enhanced signal of the tissues in which they are distributed, as fast *T*_1_ relaxation allows for the accumulation of more signal intensity using short repetition times.^7^ The efficacy of Gd(III) as a *T*_1_ relaxation agent is related to the dipolar interaction between the nuclear and electron spins, which is particularly efficient due to the presence of seven unpaired electrons and the long electronic relaxation time.^8,9^ While the use of Gd(III) CAs is regarded to be safe, a few cases of adverse effects have been reported.^10^ Long-term accumulation of Gd(III) in patients that received multiple doses was also described, which stimulated the search for alternative CAs.^11^

**Chart 1:**
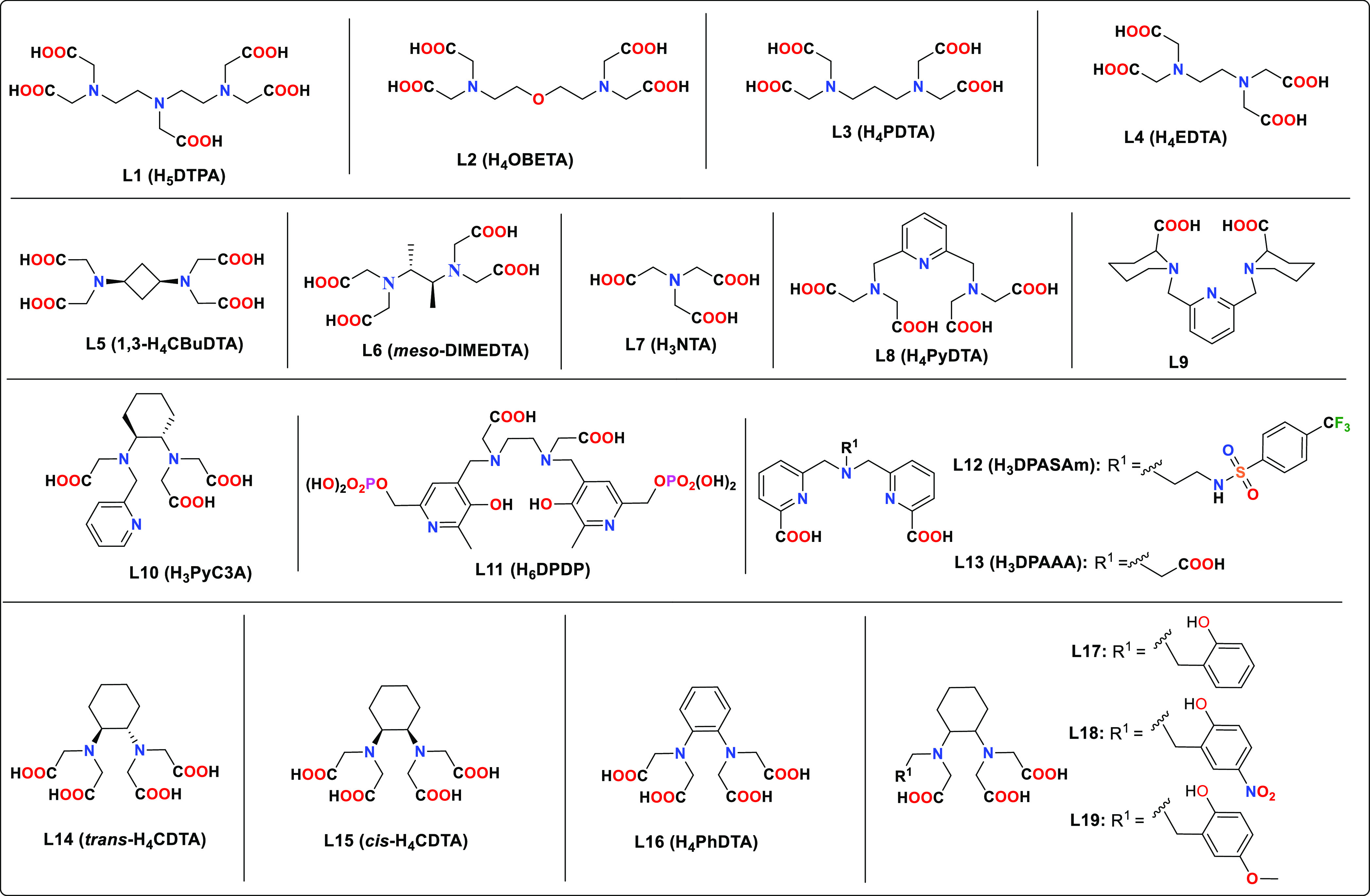
Nonmacrocyclic Ligands Discussed in the Text

High-spin Mn(II) possesses five unpaired electrons that originate a symmetrical ^6^S ground state term for the free ion. This leads to a slow electronic relaxation, making Mn(II) an efficient relaxation agent.^12^ Thus, it is not surprising that Mn(II) complexes were considered as CA candidates with the advent of MRI in the 1970s and 1980s.^13,14^ The Mn(II)-based CA [Mn(DPDP)]^4–^ (DPDP^6–^ = *N*,*N*′-dipyridoxylethylenediamine-*N*,*N*′-diacetate5,5′-bis(phosphate), **L11**, Chart 1)^15^ was also introduced in clinical practice for liver imaging,^16,17^ though its use has been discontinued due to poor sales.^18^ Nevertheless, the problems associated with Gd(III) toxicity and deposition have provoked a renewed interest in Mn(II)-based MRI contrast agents.^19^ One of the main aims of the research in this field is to develop stable and inert complexes endowed with high relaxation efficiencies.^20−29^

In a recent paper, we proposed an empirical correlation to predict and rationalize the thermodynamic stabilities of Gd(III) complexes with polyaminopolycarboxylate ligands.^30^ We showed that the thermodynamic stability constants and pGd values can be approximated to a good accuracy using structural descriptors. The contribution of each structural descriptor (ligand motif) to complex stability was obtained by a least-squares fitting procedure to stability data reported in the literature. The stability constants and pGd values were subsequently obtained by adding the contributions of each structural descriptor. We validated the predictive character of the model by determining the stability constants of a test set of complexes. The prediction of Mn(II) complex stability is of great interest to aid ligand design and reduce synthetic efforts. Thus, we envisaged to extend to Mn(II) the methodology developed to predict Gd(III) complex stabilities.

This paper presents an overview of the stability constants of Mn(II) complexes reported in the literature, which are subsequently used to develop an empirical correlation mentioned above. The contributions of the different structural motifs are then discussed and compared with those reported previously for Gd(III). To support our analysis, we also report here the X-ray structure of the Mn(II) complex of DOTA^4–^, which displays eight-coordinate Mn(II) ions.

## Results and Discussion

### Coordination Numbers in Mn(II) Complexes

**Chart 2:**
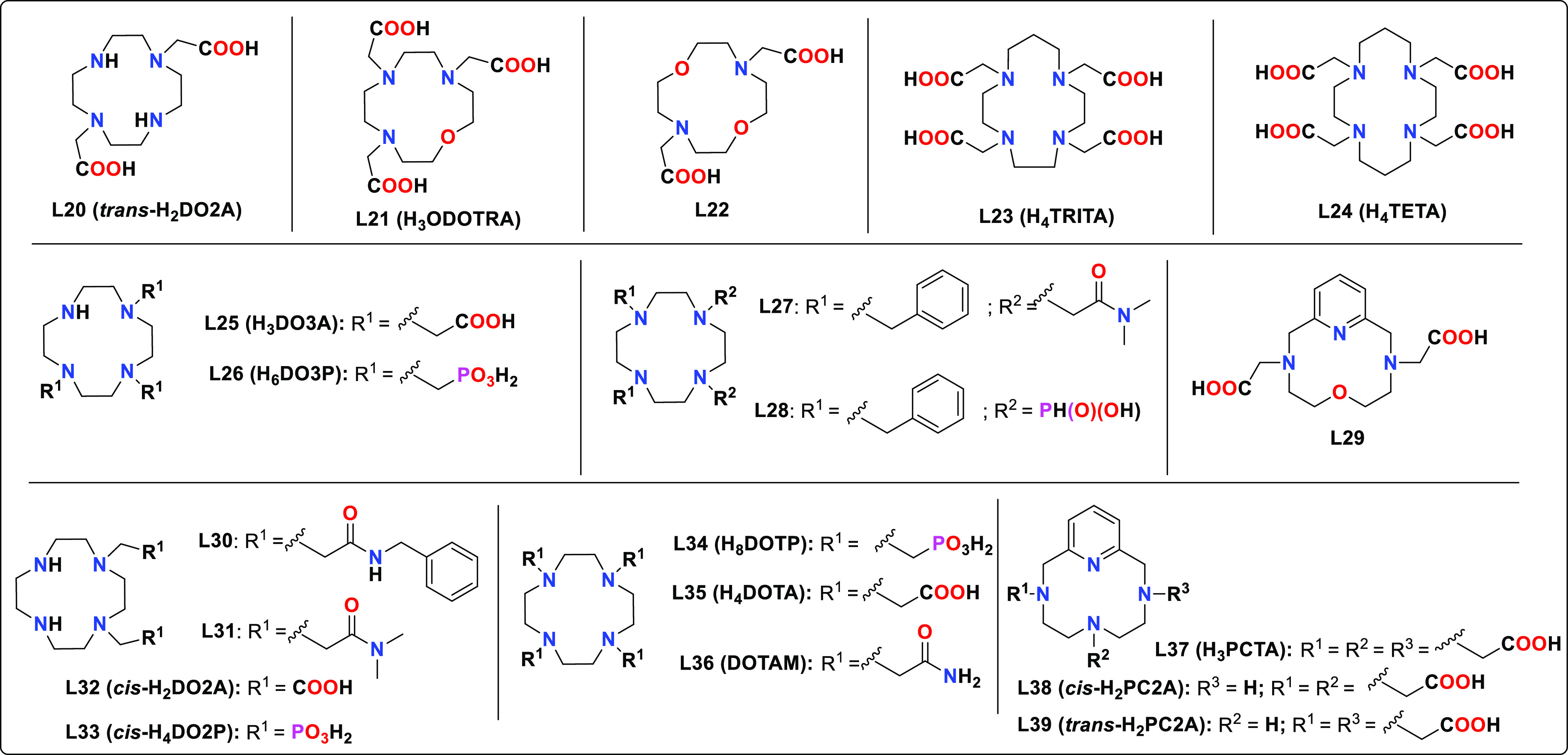
Macrocyclic Ligands Discussed in the Text

The analysis described in this work assumes that the thermodynamic stability of Mn(II) complexes can be predicted by adding the contributions of the different donor groups present in the ligand structure. However, the number of donor groups that contribute to complex stability is limited by the coordination number of the metal ion. Once the coordination sphere is saturated, the incorporation of additional donor groups into the ligand scaffold is not expected to contribute to an increased stability. The metal ion in Mn(II) complexes with polyaminopolycarboxylate ligands generally displays coordination numbers 6 or 7. Depending on the denticity of the ligand, water molecules present in the first coordination sphere may complete the metal coordination environment. Heptacoordinated metal complexes are relatively rare within the first-row transition-metal series but are more abundant for Mn(II) than for any other metal ion within the series.^31,32^ Typical seven-coordinate Mn(II) complexes are those with EDTA^4–^ (**L4**, Chart 1) and its derivatives,^33^ in which a coordinated water molecule completes the metal coordination sphere.^34−37^ A remarkable example of this class is H_3_PyC3A (**L10**, Chart 1), which forms a very stable Mn(II) complex that generates excellent MRI contrast.^38,39^ Seven coordination is also favored by 15-membered macrocyclic ligands containing five donor atoms, which generally provide pentagonal bipyramidal coordination in which the equatorial positions are occupied by the donor atoms of the macrocyclic unit.^40,41^ The Mn(II) complex with the triethyl ester derivative of the 12-membered macrocycle H_3_PCTA (**L37**, Chart 2) was also found to be seven-coordinated in the solid state.^42^ However, complexes with 1,4,7-triazacyclononane derivatives such as H_3_NOTA and related ligands generally form six-coordinate complexes in solution.^43−45^

Eight-coordinate Mn(II) complexes are rare.^46,47^ Eight-coordinate bispidine derivatives providing exceptionally high thermodynamic stabilities (log *K*_MnL_ > 24) have been reported recently.^48^ Remarkable examples in the context of MRI contrast agents are the cyclen derivatives [Mn(DOTAM)]^2+^ (DOTAM = **L36**, Chart 2) and the analogue containing four 2-pyridyl pendants, which were both found to exhibit eight-coordinate metal ions in the solid state.^49,50^ However, it is not clear whether eight coordination is favored for these complexes by the presence of charge-neutral pendant arms. The charge-neutral [Mn(*cis*-DO2A)] complex (see **L32**, Chart 2) was found to display seven-coordinate Mn(II) ions in solution, thanks to the presence of a coordinated water molecule.^51^ A seven-coordinate structure was also observed in the solid state.^52^ The incorporation of a third acetic acid pendant into the cyclen structure to give H_3_DO3A (**L25**, Chart 2) yields a seven-coordinate Mn(II) complex, as demonstrated by relaxometric studies.^51^ The coordination number of the metal ion in [Mn(DOTA)]^2–^ (see **L35**, Chart 2) was never ascertained, though the small zero field splitting evidenced by electron paramagnetic resonance (EPR) measurements is compatible with a rigid and symmetrical coordination environment.^53,54^

### Crystal Structure of [Mn_2_(DOTA)(H_2_O)_2_]

**Figure 1:**
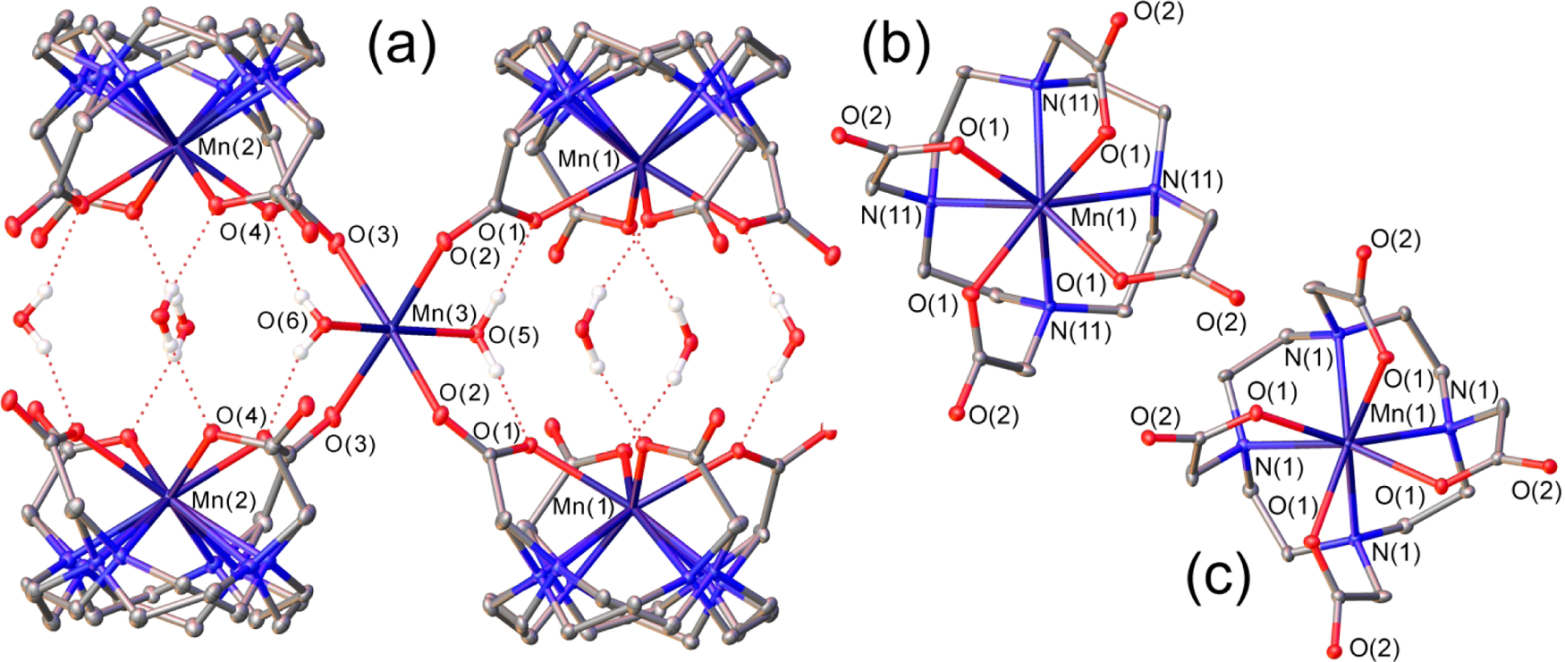
View of the crystal structure of [Mn_2_(DOTA)(H_2_O)_2_] showing the [Mn(DOTA)]^2–^ units joined by six-coordinate exocyclic Mn(II) (a), and views of the square antiprismatic (SAP) (b) and twisted square antiprismatic (TSAP) (c) isomers of [Mn(DOTA)]^2–^ complexes. Oak Ridge thermal-ellipsoid plot (ORTEP)^69^ plots are at the 30% probability level.

Crystals with formula [Mn_2_(DOTA)(H_2_O)_2_] were obtained from an aqueous solution of the [Mn(DOTA)]^2–^ complex in the presence of 1 equiv of Mn(II). The compound crystallizes in the tetragonal *P*4/*m* space group. Crystals contain [Mn(DOTA)]^2–^ entities joined by exocyclic Mn(II) ions with octahedral coordination, provided by four oxygen atoms of bridging μ_2_–η^1^:η^1^-carboxylate groups^55^ and two coordinated water molecules (Figure 1). The Mn–O distances involving the coordinated water molecules (Table 1) are close to those observed in the solid state for (NH_4_)_2_[Mn(H_2_O)_6_](SO_4_).^56^ The coordinated water molecules are involved in hydrogen bonds with oxygen atoms of the carboxylate groups.

**Table 1:** Bond Distances (Å) and Angles (deg) Observed in the Crystal Structure of [Mn_2_(DOTA)(H_2_O)_2_]

Mn(1)–O(1)	2.2867(17)	Mn(2)–O(4)	2.2845(18)
Mn(1)–N(1)	2.462(4)	Mn(2)–N(3)	2.449(3)
Mn(1)–N(11)	2.416(7)	Mn(2)–N(31)	2.398(14)
Mn(1)–N_4_,_SAP_^a^	1.218	Mn(2)–N_4_,_SAP_^a^	1.184
Mn(1)–N_4_,_TSAP_^a^	1.357	Mn(2)–N_4_,_TSAP_^a^	1.308
ϕ, SAP^b^	43.0	ϕ, SAP^b^	41.2
ϕ, TSAP^b^	26.6	ϕ, TSAP^b^	25.5
Mn(3)–O(2)	2.2154(18)	Mn(3)–O(3)	2.2153(19)
Mn(3)–O(5)	2.198(2)	Mn(3)–O(6)	2.186(3)

a Distance between the metal ion and the plane defined by the four N atoms of the macrocycle (N_4_).

b Twist angle of the O_4_ and N_4_ planes.

The macrocyclic fragment in each of the [Mn(DOTA)]^2–^ entities is disordered into two positions, which correspond to the two square [3333] conformations^57^ of cyclen that are usually denoted as (δδδδ) and (λλλλ).^58^ Interestingly, the position of the pendant arms is not disordered, adopting either a Δ or a Λ conformation for each [Mn(DOTA)]^2–^ entity.^59^ As a result, the two disordered macrocyclic units generate the Δ(λλλλ) and Δ(δδδδ) isomers, which provide square antiprismatic (SAP) and twisted square antiprismatic (TSAP) coordination polyhedra, respectively.^60,61^ An inversion center relates the Δ(λλλλ)/Λ(δδδδ) and Δ(δδδδ)/Λ(λλλλ) enantiomeric pairs of [Mn(DOTA)]^2–^. Thus, all four stereoisomers of the complex are present in the crystal lattice. The crystal lattice contains two [Mn(DOTA)]^2–^ entities with slightly different bond distances (Table 1). The twist angles (ϕ) of the O_4_ and N_4_ planes that define the square planes of the coordination polyhedra are close to those expected for SAP (45°) or TSAP (22.5°) coordination. The Mn–N distances in TSAP isomers are ∼0.05 Å longer than in the corresponding SAP isomers. As a result, the Mn(II) ions reside closer to the N_4_ plane in the SAP isomers than in the TSAP counterparts (see Mn–N_4_ distances in Table 1). All of these structural features parallel those observed for DOTA-type complexes of the lanthanide ions and Sc(III).^62−64^ The Mn–O distances are longer than those involving carboxylate oxygen atoms in seven-coordinate Mn(II) complexes (ca. 2.17–2.27 Å),^24,52,65−67^ as a result of the higher coordination number of the metal ion in [Mn(DOTA)]^2–^. A similar situation is observed for the Mn–N bonds, which fall within the range 2.33–2.45 Å for seven-coordinate complexes containing amine N atoms.^52,67,68^

### Stability Constants of Mn(II) Complexes

The literature reports a wide collection of thermodynamic stability constants determined for Mn(II) complexes with a wide variety of ligands. In this study, we aimed at predicting the stability constants of Mn(II) complexes, relevant as MRI contrast agents, using structural descriptors. Thus, we included in our study ligands that contain structural motifs present in the ligands used for this purpose. Among the nonmacrocyclic ligands, two families have been widely investigated for Mn(II) complexation. The first class comprises H_4_EDTA (**L4**, Chart 1) and its derivatives, including: (1) EDTA analogues in which the ethyl spacer is replaced by propyl (H_4_PDTA, **L3**),^70^ cyclohexyl (H_4_CDTA, **L14**),^71^ phenyl (H_4_PhDTA, **L16**),^72^ 1,2-cyclobutyl,^73^ or 1,3-cyclobutyl^74^ (H_4_CBDTA, **L5**) groups. (2) Ligands bearing one of these spacers in which some of the acetic acid arms are replaced by donor groups such as phenols (i.e., **L17**–**L19**) or pyridine groups (i.e. H_3_PyC3A, **L10**). (3) Extended H_4_EDTA structures such as those of H_4_OBETA (**L2**),^75^ H_4_PyDTA (**L8**),^76^ and H_4_EGTA.^75^ (4) Ligands related to the latter three classes in which some donor groups are absent.^76^ A more exhaustive list of ligands and their protonation and stability constants is provided in Table S1 (Supporting Information).

A second ligand family comprises tripodal ligands, in which the different donor groups, up to three, are appended on an amine nitrogen atom. This family includes H_3_NTA^77^ (**L7**) and derivatives in which acetic acid groups are replaced by different donors like picolinic acid (i.e. H_3_DPAAA, **L13**),^21^ sulphonamide (i.e. H_3_DPASAm, **L12**)^78^ or methylphosphonic acid^79^ groups. Table 2 presents the stability constants reported for Mn(II) complexes of selected nonmacrocyclic ligands.

**Table 2:** Stability Constants (log *K *_MnL_ Values, 25 °C), Values of pMn, Structural Descriptors, and Calculated log *K*_MnL_ and pMn Values for Mn(II) Complexes

	log *K*_MnL_	pMn	descriptors^e^	ref	log *K*_MnL_^calc^	pMn^calc^
**L1 (H**_**5**_**DTPA)**	15.50	12.07	3N + 5C	(102)	17.12 (14.47^a^)	11.97^a^
	14.54	11.88		(71)		
**L2 (H**_**4**_**OBETA)**	13.57	11.00	3N + 4C + SOe	(75)	12.39	11.12
**L3 (H**_**4**_**PDTA)**	10.01	7.44	2N + 4C + SProp	(103)	10.41	8.66
**L4 (H**_**4**_**EDTA)**	12.46	11.62	2N + 4C	(71)	13.18	11.50
**L5 (1,3-H**_**4**_**CBuDTA)**	10.78	9.44	2N + 4C + SCBu	(74)	11.34	9.95
**L6 (*****meso*****-DIMEDTA)**	14.10	11.20	2N + 4C + 2SCalk	(70)	13.38	11.76
**L7 (H**_**3**_**NTA)**	7.44	6.36	N + 3C	(79)	9.24	8.39
**L8 (H**_**4**_**PyDTA)**	14.13	13.39	3N + 4C + SPy	(76)	14.74	13.98
**L9**	11.37	10.83	3N + 2C + 2SCyhx + SPy	(99)	11.88	10.14
**L10 (H**_**3**_**PyC3A)**	14.14	12.29	2N + 3C + Py + SCyhx	(20)	13.26	11.87
**L11 (H**_**6**_**DPDP)**	15.10	10.34	2N + 2C + 2Phe	(15)	14.16	8.46
**L12 (H**_**3**_**DPASAm)**	13.53	11.55	N + 2Pic + Sulph	(78)	13.76	11.48
**L13 (H**_**3**_**DPAAA)**	13.19	13.91	N + C + 2Pic	(21)	13.18	13.07
**L14 (*****trans*****-H**_**4**_**CDTA)**	14.32	13.59	2N + 4C + SCyhx	(71)	14.40	12.22
**L15 (*****cis*****-H**_**4**_**CDTA)**	14.19	11.54	2N + 4C + SCyhx	(98)	14.40	12.22
**L16 (H**_**4**_**PhDTA)**	11.79	12.67	2N + 4C + SPh	(72)	11.79	11.77
**L17**	14.16	9.21	2N + 3C + Phe	(95)	13.67	9.98
**L18**	13.66	11.06	2N + 3C + PheNO2	(95)	11.86	10.17
**L19**	14.61	8.41	2N + 3C + PheOMe	(95)	13.35	8.24
**L20 (*****trans*****-H**_**2**_**DO2A)**	14.64	8.95	A_12_ + 2C	(80)	14.95	10.05
**L21 (H**_**3**_**ODOTRA)**	13.88	13.09	A_12_ + 3C + SOe	(81)	15.52	11.92
**L22**	9.38	9.32	A_12_ + 2C + 2SOe	(87)	10.79	8.51
**L23 (H**_**4**_**TRITA)**	16.74	11.41	A_12_ + 4C + Spropyl	(93)	17.48	12.49
**L24 (H**_**4**_**TETA)**	11.27	6.51	A_12_ + 4C + 2Spropyl	(93)	14.71 (12.06^b^)	7.01^b^
**L25 (H**_**3**_**DO3A)**	19.43	13.68	A_12_ + 3C	(104)	17.60	12.69
**L26 (H**_**6**_**DO3P)**	17.45	8.82	A_12_ + 3Pho	(81)	19.91 (16.49^c^)	
**L27**	11.54	7.91	A_12_ + 2A_NR2_	(96)	12.19	9.29
**L28**	9.39	6.58	A_12_ + 2Phosphi	(82)	10.13	6.59
**L29**	13.03	10.99	A_12_ + 2C + SOe + SPy	(105)	13.14	11.57
**L30**	10.72	9.34	A_12_ + 2 A_NHR_	(96)	11.49	8.91
**L31**	12.64	9.83	A_12_ + 2 A_NR2_	(96)	12.19	9.29
**L32(*****cis*****-H**_**2**_**DO2A)**	15.22	9.99	A_12_ + 2C	(52)	14.95	10.05
**L33 (*****cis*****-H**_**4**_**DO2P)**	15.41	7.41	A_12_ + 2Pho	(82)	16.49	9.15
**L34 (H**_**8**_**DOTP)**	18.98	8.64	A_12_ + 4Pho	(81)	23.33 (16.49^c^)	
**L35 (H_4_DOTA)**	19.44	13.95	A_12_ + 4C	(104)	20.25	15.33
**L36 (DOTAM)**	11.96	12.65	A_12_ + 4A_NH2_	(81)	12.01	12.01
**L37 (H**_**3**_**PCTA)**	16.83	15.13	A_12_ + 3C + SPy	(81)	17.87	14.70
**L38 (*****cis*****-H**_**2**_**PC2A)**	15.53	12.15	A_12_ + 2C + SPy	(86)	15.22	12.06
**L39 (*****trans*****-H**_**2**_**PC2A)**	17.09	13.18	A_12_ + 2C + SPy	(86)	15.22	12.06
**L40**	10.61	6.35	A_9_ + 2Pho + SOe	(88)	10.55	6.39
**L41**	4.30	6.09	A_9_ + 2Phosphi + SOe	(88)	4.19	d
**L42 (H**_**2**_**NO2A)**	11.56	8.02	A_9_ + 2C	(106)	11.09	8.06
**L43**	7.73	6.13	A_9_ + 2C + SOe	(107)	9.01	7.29
**L44 (H**_**4**_**AAZTA)**	14.19	12.52	A_AAZTA_ + 4C	(92)	13.89	12.01
**L45**	11.00	9.10	A_AAZTA_ + 3C	(92)	11.24	9.37
**L46**	10.67	8.72	A_AAZTA_ + 3Cα	(92)	10.91	8.50
**L47**	10.85	7.03	A_15_	(101)	10.88	6.55
**L48**	11.09	6.92	A_15_ + 2SCalk	(101)	11.08	6.77
**L49**	10.89	8.67	A_15_ + SPy	(40)	11.15	8.56
**L50**	7.18	6.40	A_15_ + 2SOe + SPy	(40)	6.99	7.02

a Calculated for 3N + 4C.

b Value calculated for A_12_ + 4C + 2Spropyl.

c Calculated for A_12_ + 2Pho.

d Excluded from the fit because the complex is nearly fully dissociated under the conditions used to define pMn.

e Descriptors detailed in Table 3.

**Chart 3:**
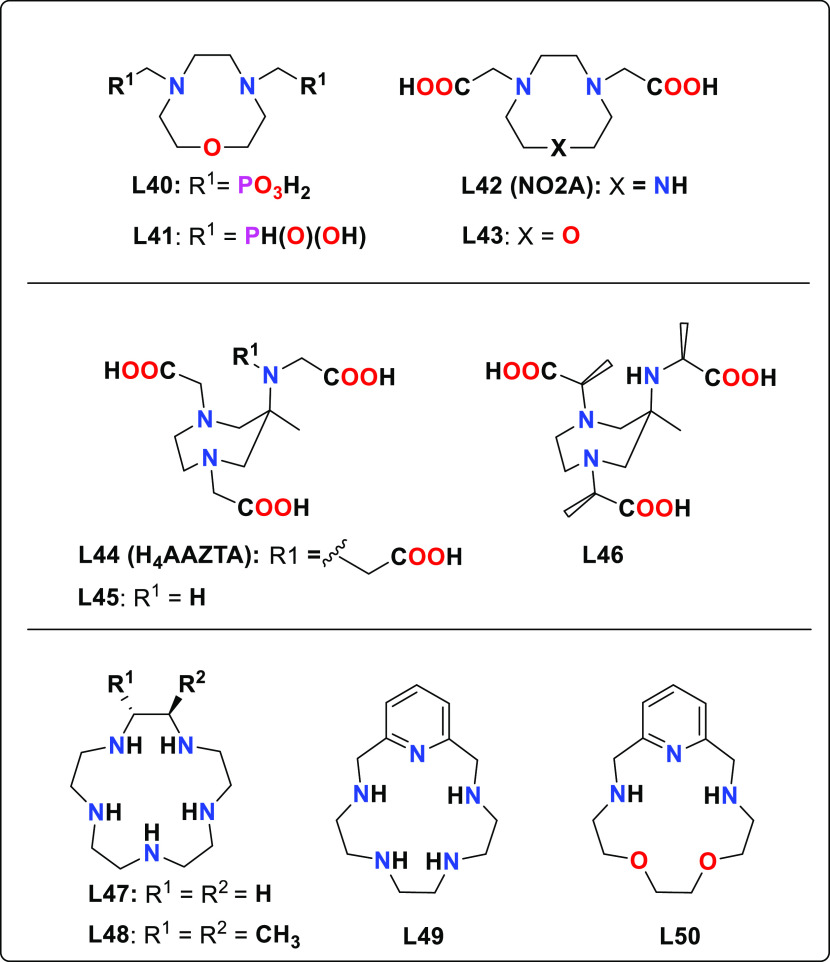
Representative Examples of Ligands Derived from TACN, AAZTA, and 15-Membered Macrocycles

The class of macrocyclic ligands that have been more extensively investigated for Mn(II) complexation is certainly the family of tetraazamacrocycles (Chart 2), more commonly cyclen (1,4,7,10-tetraazacyclododecane),^80−82^ cyclam (1,4,8,11-tetraazacyclotetradecane),^83,84^ or pyclen (3,6,9,15-tetraazabicyclo[9.3.1]pentadeca-1(15),11,13-triene)^26,85,86^ functionalized with different pendant arms, typically acetic acid, primary or N-substituted acetamides, methylphosphonic, methylphosphinic, or picolinic acid groups, among others. Some of these macrocycles incorporate ether oxygen atoms into the macrocyclic structure replacing some of the amine N atoms.^87^ Alternatively, macrocyclic ligands derived from 1,4,7-triazacyclononane (TACN) functionalized with different pendant arms, and often with mixed N/O donor sets in the macrocyclic scaffold, can be used as ligands for Mn(II) (i.e. **L43**, Chart 3).^88−90^ The structurally related 15-membered macrocyclic ligands containing mixed N/O donor sets form rather stable complexes as well (i.e. **L47**–**L50**, Chart 3).^40,41^ Macrocyclic ligands of this family incorporating acetic acid pendant arms were also investigated for Mn(II) complexation.^91^ Finally, the stability of Mn(II) complexes with a few mesocyclic ligands derived from AAZTA was also explored (i.e. **L44**–**L46**, Chart 3).^92^

**Figure 2:**
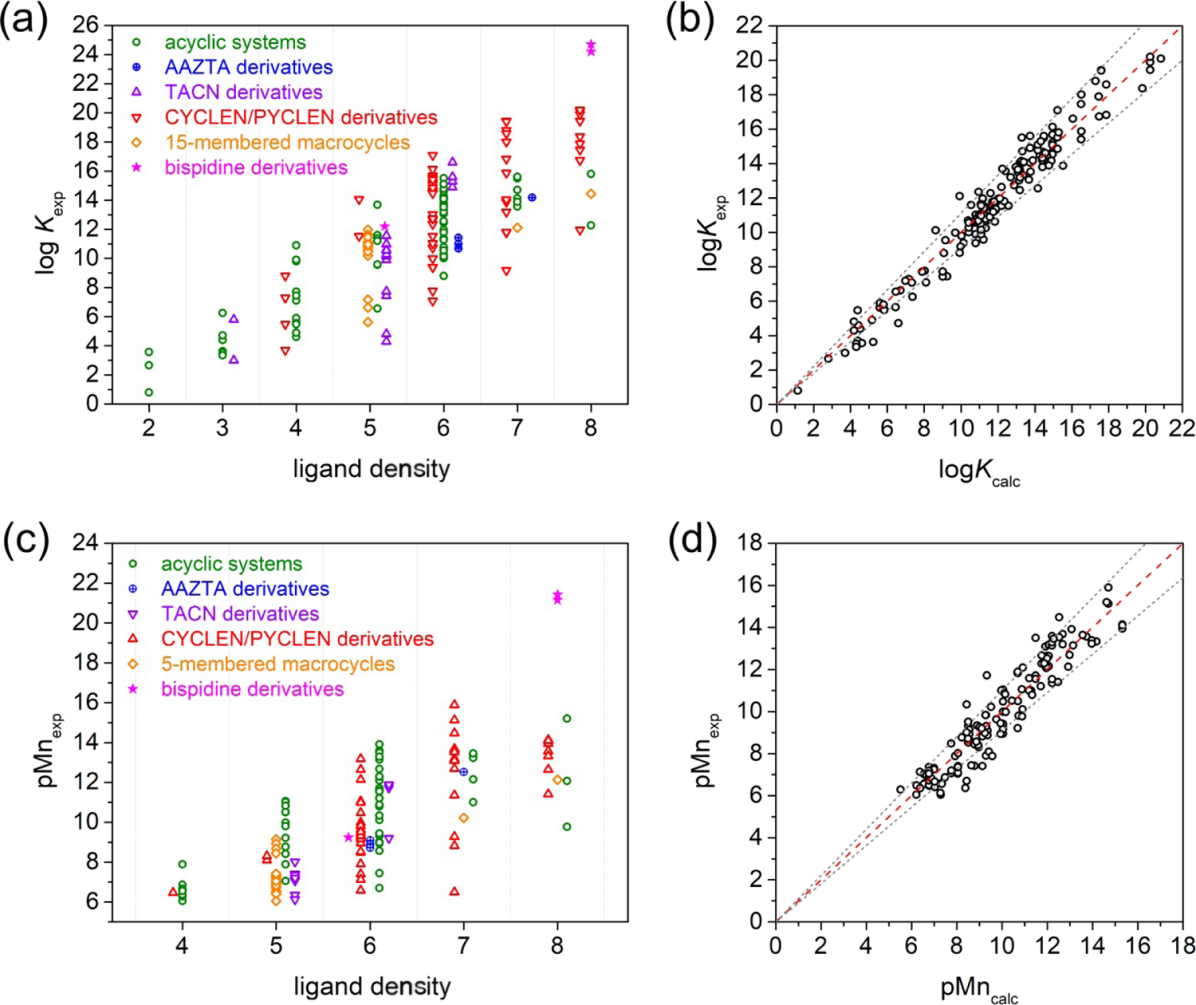
(a) Stability constants (log *K*_MnL_ values) and (c) pMn values of Mn(II) complexes classified according to ligand denticity for different structural families. (b) Plot of the log *K*_MnL_ values reported in the literature (168 values) versus those calculated using eq 1 and (d) plot of pMn values (141 values) versus those calculated using eq 2. Dashed lines represent the lines of identity, while the area within gray dotted lines corresponds to deviations <±10% between experimental and calculated values.

The thermodynamic stability of Mn(II) complexes depends on several factors, as illustrated in Figure 2. Stability constants generally increase with increasing ligand denticity, as would be expected. The highest log *K*_MnL_ values are observed for complexes with hepta- and particularly octadentate ligands. This suggests that several Mn(II) complexes display coordination number eight in solution, as, for instance, the bispidines reported recently by Comba^48^ and some cyclen derivatives such as [Mn(DOTA)]^2–^. In the latter case, stability constants of log *K*_MnL_ = 20.2 and 19.9 were determined using ionic strengths of 0.1 M Me_4_N(NO_3_)^93^ and Me_4_NCl,^52^ while that reported for [Mn(DO3A)]^−^ in 0.1 M Me_4_NCl is slightly lower (log *K*_MnL_ = 19.4).^52^ The log *K*_MnL_ values determined for a given ligand denticity spread over several orders of magnitude, highlighting the critical effect of ligand topology and the nature of the donor groups incorporated into the ligand scaffold. The data shown in Figure 2 also evidence that macrocyclic ligands derived from the 12-membered macrocycles pyclen, and particularly cyclen, tend to form Mn(II) complexes with higher stabilities than other ligand classes, with the exception of the bispidine ligands described recently,^48^ which form extraordinary stable Mn(II) complexes. Taken together, the stability constants reported for Mn(II) complexes span more than 20 orders of magnitude, from log *K*_MnL_ values of ca. 1–3 for simple bidentate ligands (i.e. picolinic acid)^94^ to log *K*_MnL_ ∼ 24 for the mentioned bispidine complexes.

### Structural Descriptors

The descriptors used to predict the thermodynamic stability of Mn(II) complexes are essentially those used previously for Gd(III)^30^ and are listed in Table 3. Linear polyaminopolycarboxylate ligands are described by the corresponding number of amine N atoms, denoted as N, and the number of acetic acid groups C. For instance, H_4_EDTA is described as *n*N = 2 + *n*C = 4, where *n* indicates the number of groups of a given class. In the case of macrocyclic ligands, the macrocyclic unit as a whole, including the donor atoms, is represented by a single descriptor denoted as A_9_, A_12_, and A_15_ for triaza-, tetraaza-, and pentaaza-macrocycles, respectively. These descriptors are intended to catch the peculiarities of each macrocyclic unit in terms of not only the number of donor atoms but also the match between the size of the cavity and that of the Mn(II) ion. Thus, H_4_DOTA is described as A_12_ + 4C, while H_2_NO2A is defined as A_9_ + 2C. Similarly, we used a descriptor A_AAZTA_ to account for the ligand 6-amino-6-methylperhydro-1,4-diazepine fragment. Thus, H_4_AAZTA (**L44**, Chart 3) is described as A_AAZTA_ + 4C. Bispidine derivatives were excluded from the analysis presented below due to the scarce thermodynamic data reported for this family of complexes.

**Table 3:** Structural Descriptors Used for the Prediction of Mn(II) Complex Stability

N	amine N atom
Pho	methylphosphonic acid
Phosphi	methylphosphinic acid
C	acetic acid
HE	hydroxyethyl
C_α_	α-alkyl acetic acid
A_NH2_	primary acetamide
A_NHR_	secondary acetamide
A_NR2_	tertiary acetamide
Pic	2-methylpicolinic acid
Phe	2-methylphenol
PheNO2	2-methyl-4-nitrophenol
PheOMe	2-methyl-4-methoxyphenol
Sulph	ethylsulphonamide
Py	2-methylpyridine
SCalk	C-alkyl substituent
SOe	ether O atom
Spropyl	propyl group
SCyhx	cyclohexyl ring
SPh	phenyl ring
SPy	pyridyl ring
SCybu	cyclobutyl ring
A_9_	triazacyclononane ring
A_12_	tetraazacyclododecane ring
A_15_	pentaazacyclopentadecane ring
A_AAZTA_	6-amino-6-methylperhydro-1,4-diazepine moiety

The different donor groups incorporated into linear or macrocyclic structures are associated with the following descriptors: methylphosphonic acid (Pho), methylphosphinic acid (Phosphi), hydroxyethyl (HE), 2-methypyridine (Py), primary acetamide (A_NH2_), secondary acetamide (A_NHR_), tertiary acetamide (A_NR2_), 2-propionic acid or α-substituted acetic acid (C_α_), 2-methylpicolinic acid (Pic), and ethylsulphonamide (Sulph). The stability constants reported for 2-methylphenol derivatives appear to be very sensitive to the nature of the substituent at position 4. Indeed, the stability constants of derivatives containing the electron withdrawing −NO_2_ substituent (i.e. **L18**, Chart 1) are lower than those of unsubstituted derivatives (**L17**), which in turn are lower than the stability of derivatives containing an electron-donating −OMe substituent (**L19**, see stability constants in Table 2).^95^ We thus used three structural descriptors for 2-methylphenol groups, denoted as Phe, PheNO2, and PheOMe. Similarly, the use of different descriptors for primary (A_NH2_), secondary (A_NHR_), and tertiary (A_NR2_) acetamides is justified by the stability constants of complexes with ligands such as **L30**, **L31**,^96,97^ and pyclen derivatives with amide groups,^81^ which indicate that complex stability increases upon increasing the number of alkyl substituents. Ligands containing α-substituted acetic acid arms generally provide Mn(II) complexes with slightly lower stability than the parent derivatives (i.e. **L45** and **L46**),^92^ and thus we used two different descriptors to consider the effect of α-substitution.

The comparison of the stability constants reported for H_4_EDTA derivatives bearing different spacers evidences that this structural modification has an important impact on the thermodynamic stability constants. The incorporation of a cyclohexyl ring (i.e. H_4_CDTA, **L14**) results in increased stability, while all remaining modifications result in lower log *K*_MnL_ values than for the parent complex. We note that the use of 1,2-cyclobutyl or *cis*-1,3-cyclobutyl spacers yields complexes with very similar stabilities.^73,74^ Thus, these structural modifications, consisting in replacing an ethyl group such as that in H_4_EDTA by a cyclobutyl ring, are described by the same structural descriptor SCybu. Similarly, the complexes of *cis*- and *trans*-H_4_CDTA give also very similar values of log *K*_MnL_,^98^ and thus these structural modifications are described by a single structural descriptor SCyhx. Additionally, the same descriptor was used to account for the incorporation of a piperidine ring into the ligand scaffold (see **L9**, Chart 1).^99^ Similarly, the incorporation of a phenyl ring (i.e. H_4_PhDTA, **L16**) and a propyl chain (i.e. H_4_PDTA, **L3**) are denoted as SPh and Spropyl, respectively. The same descriptors are employed to account for the introduction of phenyl or propyl groups into macrocyclic units, for instance, the propyl chains in H_4_TRITA (**L23**) and H_4_TETA (**L24**).^93^

A rather common structural modification introduced to macrocyclic systems consists in replacing amine N atoms by ether oxygen atoms (**L21**,^81^**L22**,^87^**L40**,^88^**L41**,^88^**L50**^40^) or pyridyl rings (i.e. all pyclen derivatives, **L49** and **L50**).^40^ These structural modifications are considered by structural descriptors SOe and SPy. Some nonmacrocyclic ligands also incorporate these structural motifs, for instance, H_4_OBETA (**L2**)^75,100^ and H_4_PyDTA (**L8**).^76^

The log *K*_MnL_ values of C-alkylated linear and macrocyclic complexes, such as *meso*-H_4_DIMEDTA (**L6**)^70^ and **L48**,^101^ are slightly higher than the parent nonsubstituted derivatives. Thus, this alteration was considered with an additional descriptor (SCalk).

### Prediction of Stability Constants and Conditional Stability

The structural descriptors presented in the previous section were used to estimate the log *K*_MnL_ values of Mn(II) complexes using the following expression
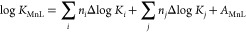
1

In this expression, *n*_*i*_ is the number of structural descriptors of type *i*, while Δlog *K*_*i*_ represents the contribution to the stability constant of this donor group. The second term accounts for the different structural modifications present in the ligand structure, where *n*_*j*_ is the number modifications of a given type and Δlog *K*_*j*_ its contribution to complex stability. For the complexes with acyclic ligands, *A*_MnL_ was set to zero.

Similarly, we used a similar expression to analyze complex stabilities at pH 7.4 using pMn values (eq 2), which are defined here as −log[Mn]_free_ for a total Mn(II) concentration of 1 μM and a total ligand concentration of [L]_tot_ = 10 μM.
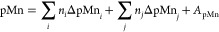
2

The values of pMn allow for a comparison of the stabilities of complexes with different ligands at physiological pH, which depend not only on log *K*_MnL_ values but also on ligand basicity as well. In principle, one could define pMn using different conditions, for instance, taking equimolar concentrations of ligand and metal ions. We have chosen here the conditions proposed by Raymond,^115^ which imply using a 10-fold ligand excess. This results in higher pMn values, increasing the number of ligands that provide a pMn value >6, as pMn = 6 corresponds to a fully dissociated system. Figure 2 shows that the pMn values vary in the range of ca. 6–21, and tend to increase with ligand denticity. Bidentate and tridentate ligands generally provide pMn values of 6 for the definition used here, and thus these ligands were excluded in Figure 2c.

A least-squares fit of 168 log *K*_MnL_ values reported in the literature to eq 1 provided the contribution to the complex stability of the different structural descriptors, which were used as fitting parameters. The results of the analysis are provided in Table 4. The agreement between the experimental log *K*_MnL_ values and those predicted by eq 1 is very good, as shown in Figure 2. The linear fit of the data provides a slope very close to 1 [0.997(5)], as would be expected, with a Pearson’s correlation coefficient of 0.9978. The mean deviation of the calculated data with respect to the experimental values is only 0.63. The agreement between the experimental and calculated data is remarkable, as the mean deviation is comparable to the differences in stability constants reported for the same system by independent groups, often using different ionic strengths. For instance, log *K*_MnL_ values differing by more than 1.3 and 0.7 log *K* units were reported for H_5_DTPA (**L1**)^108^ and H_4_EGTA.^71,75^ Similar differences in stability constants were observed for macrocyclic ligands when using different ionic strengths.^80^ Furthermore, the structural descriptors presented above predict identical log *K*_MnL_ values for regioisomeric ligands such as *cis*-H_2_PC2A (**L38**) and *trans*-H_2_PC2A (**L39**), which were found to differ by ∼1.5 log *K* units.^86^ Thus, it is obvious that the stability constants of a given complex are affected not only by the nature of the donor groups present in the ligand scaffold but also by the arrangement of these donor groups in the ligand structure.

**Table 4:** Contributions of the Different Structural Descriptors to log *K*_MnL_ and pMn Obtained from the Least-Squares Fit of the Stability Data to Equations 1 and 2 and Total Number of Structural Descriptors of Each Type (*Σn*_*i*,*j*_)^a^

	Δlog *K*_*i*,*j*_	*Σn*_*i*,*j*_	ΔpM*n*_*i*,*j*_	Σ*n*_*i*,*j*_
N	1.29(0.10)	136	0.47(0.18)	110
Pho	3.42(0.15)	23	2.19(0.18)	16
Phosphi	0.24(0.28)	6	0.91(0.48)	2
C	2.65(0.06)	286	2.64(0.10)	256
HE	–0.15(0.32)	6	0.46(0.66)	2
C_α_	2.54(0.16)	13	2.35(0.19)	10
A_NH2_	0.59(0.19)	7	1.81(0.20)	7
A_NHR_	0.92(0.26)	5	2.07(0.27)	5
A_NR2_	1.27(0.21)	9	2.26(0.23)	9
Pic	4.62(0.19)	19	4.98(0.21)	17
Phe	3.14(0.24)	9	1.12(0.26)	9
PheNO2	1.33(0.65)	2	1.31(0.67)	2
PheOMe	2.82(0.65)	2	–0.62(0.67)	2
Sulph	3.23(0.54)	3	1.05(0.56)	3
Py	1.51(0.12)	24	2.29(0.16)	15
SCalk	0.10(0.14)	39	0.11(0.15)	36
SOe	–2.08(0.14)	34	–0.77(0.16)	27
Spropyl	–2.77(0.18)	25	–2.84(0.26)	9
SCyhx	1.22(0.27)	12	0.72(0.29)	12
SPh	–1.39(0.47)	4	0.27(0.49)	4
SPy	0.27(0.22)	30	2.01(0.24)	27
SCybu	–1.84(0.44)	5	–1.55(0.46)	5
A_9_	5.79(0.26)	17	2.78(0.34)	12
A_12_	9.65(0.22)	59	4.77(0.30)	47
A_15_	10.88(0.31)	24	6.55(0.34)	23
A_AAZTA_	3.29(0.50)	4	1.45(0.57)	4

a Structural descriptors detailed in Table 3.

The analysis of the log *K*_MnL_ values using eq 1 allows us to infer the coordination number of the metal ion in certain complexes, for which the assignment of a given coordination number is ambiguous. For instance, a log *K*_MnL_ value of 17.12 is calculated for H_5_DTPA (**L1**) assuming octadentate binding of the ligand to the Mn(II) ion (3N + 5C). However, the value calculated for a seven-coordinate complex (3N + 4C) of 14.47 is in much better agreement with the experimental values of 15.50^102^ and 14.54.^71^ Thus, this complex is very likely heptacoordinated in aqueous solution, as observed in the solid state for bis(amide) derivatives of H_5_DTPA.^109^ A similar situation is observed for H_4_TETA (**L24**), for which the log *K*_MnL_ value estimated assuming eight coordination (14.71), differs considerably from the experimental value of log *K*_MnL_ = 11.27.^93^ A considerably better agreement is observed by assuming the formation of a heptacoordinated complex (Table 2). Most likely the propyl chains present in the ligand structure introduce some steric hindrance around the metal ion, favoring a lower coordination number in comparison with H_4_DOTA, as observed for the corresponding Gd(III) complexes.^110^ The presence of bulky methylphosphonic acid groups in H_6_DO3P (**L26**) and H_8_DOTP (**L34**) appears to favor the formation of six-coordinate complexes in solution (Table 2).^81^

The empirical expression obtained here may be useful to aid experimental stability constant determination, as the predicted log *K*_MnL_ value can help anticipate the pH range in which complex dissociation is expected to occur. Furthermore, eq 1 can be used to identify stability constant values that are likely to be incorrect. For instance, a stability constant of log *K*_MnL_ = 14.29 was reported for a pentadentate ligand containing a piperazine ring functionalized with a picolinic acid and an acetic acid function.^25^ The stability constant predicted with eq 1 using 2N + 1C + 1Pic + 1SCyhx is 11.07. The very large discrepancy between the experimental and calculated values suggests that the experimental stability constant may be incorrect and should be taken with some caution.

The pMn values obtained from 141 complexes were fitted to eq 2 following the same strategy used for stability constants. The number of data points used in this analysis is lower than for log *K*_MnL_, as ligands with pMn ∼ 6 had to be excluded from the analysis, and for a few systems, ligand protonation constants were not reported together with stability constants. The agreement between the experimental and calculated pMn data is reasonably good (Figure 2), though not as good as for log *K*_MnL_ values. The linear fit of the data gives a slope of 0.993(7) and a Pearson’s correlation coefficient of 0.9967. The mean deviation of calculated versus experimental data amounts to 0.66 pMn units. We note that the Δlog *K*_*i*,*j*_ and ΔpM*n*_*i*,*j*_ contributions characterizing some structural descriptors were obtained with rather large standard deviations (Table 4). This situation is generally associated with structural motifs that have been seldom incorporated into ligand structures (low Σ*n*_*i*,*j*_ values in Table 4).

**Figure 3:**
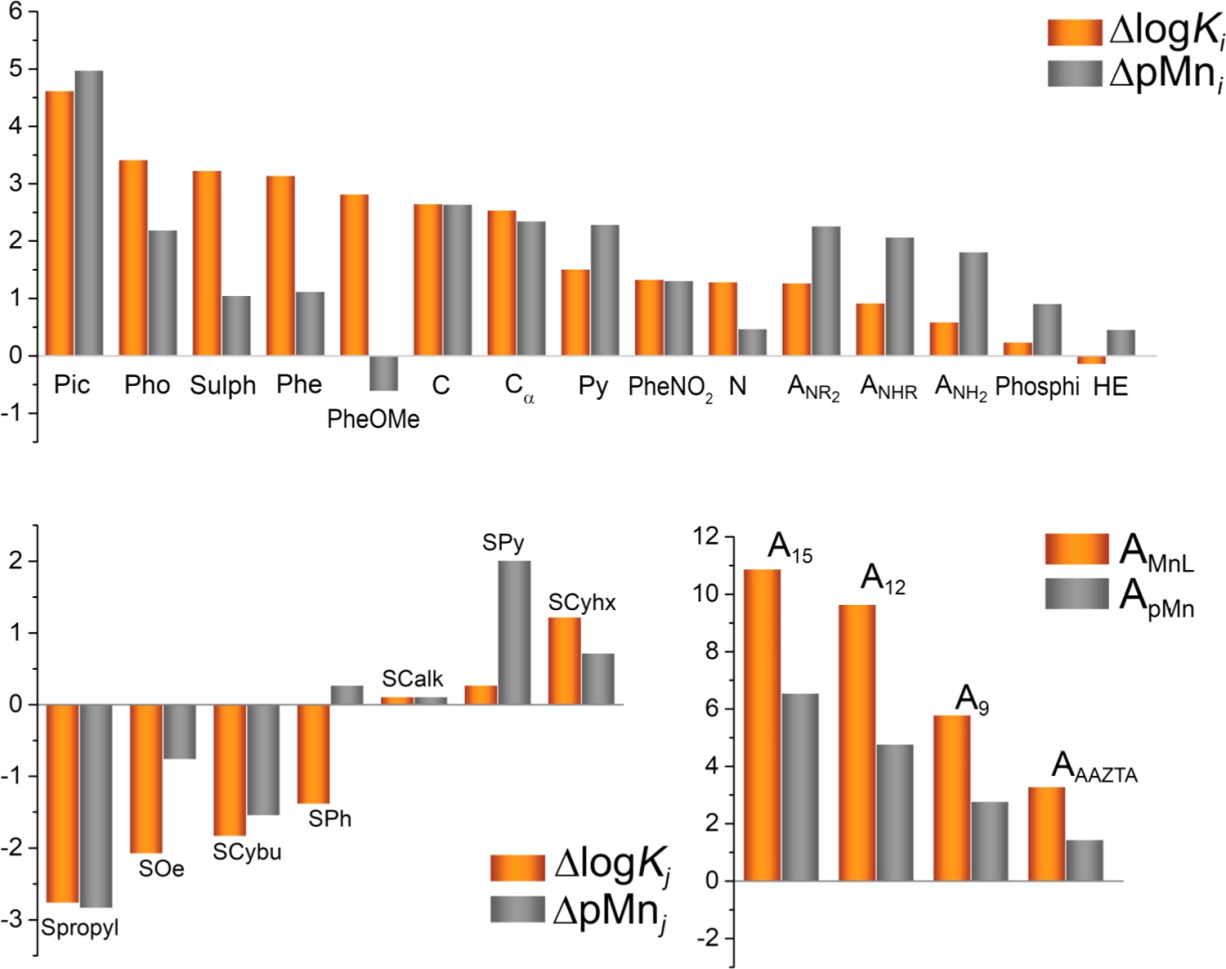
Comparison of the contributions of the different structural descriptors to log *K*_MnL_ and pMn, obtained from the least-squares fit of the stability data to eqs 1 and 2. Structural descriptors detailed in Table 3.

### Analysis of the Structural Descriptors

The contributions of the different structural descriptors to log *K*_MnL_ and pMn provide valuable information that can be used for ligand design. Figure 3 shows the contributions of the different motifs to log *K*_MnL_ and pMn. The group with the highest contribution to log *K*_MnL_ is the picolinic acid moiety (Pic), which is characterized by a Δlog *K_i_* value of 4.65. The latter value is significantly higher than the sum of the contributions of an acetic acid (C) and a pyridyl group (Py), which amounts to 4.16. Thus, picolinate units are particularly well suited for stable Mn(II) complexation. Other groups characterized by large Δlog *K_i_* contributions are methylphosphonic acid (Pho), ethylsulphonamide (Sulph), and 2-methylphenol groups, either unsubstituted at position 4 (Phe) or bearing a methoxy substituent (PheOMe). However, the high basicity of these groups results in a very significant decrease in their contribution to pMn compared with log *K*_MnL_. In other words, these groups provide a large contribution to the stability constant, but they are also prone to protonation at pH 7.4, which has a very negative impact on the stability of the complex close to physiological pH. Indeed, very high protonation constants were determined for Mn(II) complexes containing methylphosphonate,^82^ ethylsulphonamide,^78^ and phenolate groups,^95^ with log *K*_MnL_ typically >5.5. This effect is particularly dramatic for PheOMe, which is characterized by Δlog *K_i_* = 2.82 and ΔpMn*_i_* = −0.62. The lower basicity of the phenol group functionalized with a −NO_2_ substituent at position 4 results however in very similar Δlog *K_i_* and ΔpMn*_i_* values. The high basicity of amine N atoms (N) also justifies the fact that Δlog *K_i_* > ΔpMn*_i_*.

The low basicity of acetate groups (C) results in very similar Δlog *K_i_* and ΔpMn*_i_* contributions. The introduction of α-alkyl groups (C_α_) has a very minor impact in terms of Δlog *K_i_*, but decreases slightly ΔpMn*_i_*, likely because of an enhanced basicity associated with the electron-donating effect of the alkyl substituent. Picolinate groups are known to decrease the overall ligand basicity compared with similar ligands containing acetate groups, explaining that Δlog *K_i_* < ΔpMn*_i_* in the latter case.^111^

Donor groups with low basicities are generally characterized by Δlog *K_i_* < ΔpMn*_i_*, and thus are well suited to increase complex stability at physiological pH. As a result, donor groups such as 2-methylpyridine (Py) and tertiary (A_NR2_) and secondary (A_NHR_) acetamides provide contributions to ΔpMn*_i_* approaching that of carboxylates (C). The contribution of a picolinate group (4.98) is nearly identical to the sum of the contributions of acetate (2.64) and pyridine (2.29).

Concerning the effect of structural modifications, the incorporation of propyl groups (Spropyl), ether oxygen atoms (SOe), or cyclobutyl (SCybu) groups has a very negative impact on both log *K* and ΔpMn, as evidenced by their negative contributions. Replacing ethylene groups of the ligand backbone by phenyl groups has a negative impact in terms of log *K*, but results in a slight positive contribution to pMn. The incorporation of a pyridyl group into the ligand scaffold results in improved stability, with a particularly positive effect on pMn. This can be attributed to the lower basicity of pyridine with respect to amine N atoms. Examples of ligands that exploit this effect for stable Mn(II) complexation are H_4_PyDTA (**L8**) derivatives^76^ and H_2_PC2A derivatives **L38** and **L39**.^86^ Cyclohexyl rings have also a beneficial impact on complex stability when replacing ethyl groups of polyaminopolycarboxylate ligands, an effect exploited in the well-known H_3_PyC3A ligand (**L10**), which affords a stable Mn(II) complex with appealing properties as an MRI contrast agent.^38,39^

The terms describing the contributions of macrocyclic and mesocyclic platforms indicate that 15-membered macrocycles provide the largest contribution to both complex stability and pMn, followed by 12-membered macrocycles, TACN and AAZTA derivatives. The same trend is observed for both log *K* and ΔpMn values. However, one has to consider that these structural motifs contain a different number of donor groups, and thus impose some limitations to the number of additional donor atoms that can be incorporated into the Mn(II) coordination sphere. 15-Membered macrocycles generally favor seven-coordinate complexes, where two additional donor atoms coordinate to the metal ion from different sides of the macrocyclic mean plane. As a result, only one additional donor atom can be incorporated into the ligand scaffold if an inner-sphere water molecule should be present. The TACN unit contains three donor atoms, but Mn(II) complexes based on this platform do not exceed coordination number six, which greatly limits the stability that can be achieved (Figure 2). Thus, 12-membered macrocycles appear to be the best choice among those analyzed here, as they combine rather large *A*_MnL_ and *A*_pMn_ contributions and coordination numbers of seven or even eight in the case of cyclen derivatives, as demonstrated here for [Mn(DOTA)]^2–^.

**Figure 4:**
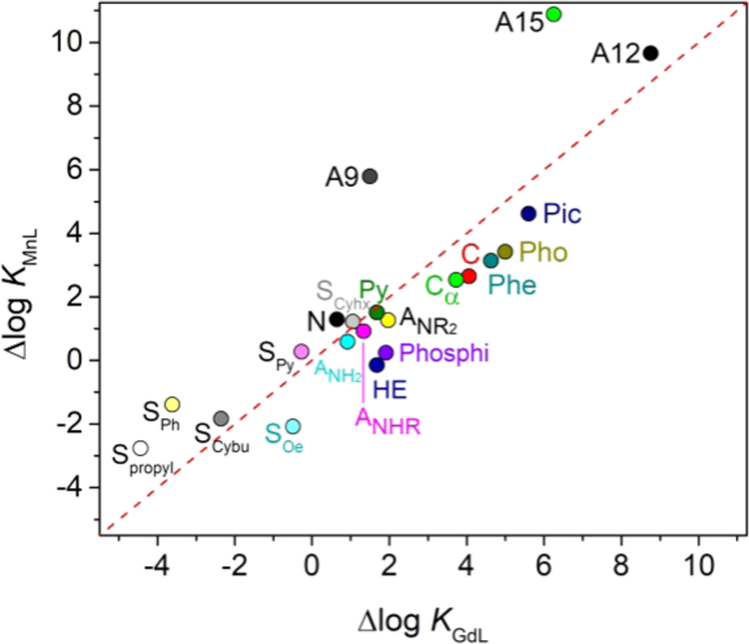
Comparison of the contributions of the different structural descriptors to the stability constants of Gd(III) and Mn(II) complexes. Data above the dashed line provide more favorable contributions to Mn(II) complex stability than to Gd(III).

Figure 4 provides a comparison of the contributions of the different structural descriptors to Mn(II) and Gd(III)^30^ complex stabilities. The Δlog *K* values of most structural descriptors fall close to the line of identity, indicating that they contribute to a similar extent to Gd(III) and Mn(II) complex stability. However, Gd(III) complexes with high denticity ligands (8–10) are often characterized by higher stability constants than the Mn(II) analogues, due to the higher coordination numbers that the former achieve. 9-Membered and 15-membered macrocyclic units appear to be better suited for Mn(II) than Gd(III) complexation, while 12-membered macrocycles provide similar contributions to the stabilities of complexes with the two metal ions. Hydroxyethylmethyl and phosphinic acid arms are not adequate for stable Mn(II) complexation. On the contrary, phenyl and propyl spacers are more detrimental to Gd(III) complex stability compared with Mn(II). In the case of Spropyl term, this is related to the strong preference of large metal ions to form five-membered chelate rings.^112^

## Conclusions

The reemergence of Mn(II) complexes as MRI contrast agents has stimulated a great amount of work to determine their thermodynamic stabilities.^48,86,95^ Compared to Gd(III)-based MRI contrast agents, the stability constants of Mn(II) complexes are typically lower. High-spin Mn(II) has no ligand field stabilization energy (LFSE), is generally kinetically labile, and forms less stable complexes than biologically important divalent cations like Fe(II), Zn(II), and Cu(II). Thus, for in vivo applications, it is critical to achieve as high a stability as possible to minimize the risk of Mn(II) dissociation in the body. This is also true for emerging applications involving Mn-52 PET imaging.^113,114^ Concurrently, Mn-based MRI contrast agents require the presence of an inner-sphere water ligand to increase the relaxivity of the complex, and leaving a coordination site vacant for water access may result in lower stability. Finally, the large size of the Mn(II) ion and lack of LFSE results in coordination numbers of 6, 7, and 8 for complexes in aqueous solution, and coordination number is difficult to predict a priori.

Here, we utilized the large body of published stability constant data to establish quantitative structure–stability correlations to predict stability constants, employing the methodology we initially applied to Gd(III). The empirical relations presented here are remarkably accurate despite known differences in experimental stability constants arising from the use of different ionic media and ionic strengths. An interesting result from this study was the prediction of the denticity of the coordinated ligand when multidentate ligands are used, e.g., [Mn(DOTA)]^2–^ has eight-coordinate Mn(II) (confirmed by X-ray crystallography), while the predicted stability constant for [Mn(DTPA)]^3–^ is consistent with seven-coordinate Mn(II). Our approach also serves to identify published stability constant data that may be incorrect. For the development of novel Mn(II) complexes, this method allows the prediction of stability constants and the ability to rule out the synthesis of likely inferior complexes. Finally, the quantification of different structural parameters like donor atom or chelate ring type allows the design of new complexes that might have optimal stability.

Having now applied this methodology to Gd(III) and Mn(II) complexes, it is apparent that this work can be extended to other metal ions for which exist a large body of stability constant data. A limitation of this work is that the method requires stability constant data for specific donors or ligand archetypes. As such, it does not anticipate novel chelators like bispidines.^27,48^ However, these types of ligands can be added to the model once stability constant data are collected for a few examples.

## Supporting Information Available

Ligand protonation constants and stability constants of Mn(II) complexes and crystallographic data (PDF)
