# Supplementary material for: Thermodynamic Stability of Mn(II) Complexes with Aminocarboxylate Ligands Analyzed Using Structural Descriptors
Source: Inorg Chem. 2022 Aug 22;61(35):14173–86. doi: 10.1021/acs.inorgchem.2c02364 (PMC9455602; doi:10.1021/acs.inorgchem.2c02364)
Supplement: Supplementary file 1 — ic2c02364_si_001.pdf [file ic2c02364_si_001.pdf]

Supporting Information

Thermodynamic stability of Mn(II) complexes with aminocarboxylate ligands analyzed using structural descriptors

Rocío Uzal-Varela,[†] Francisco Pérez-Fernández,[†] Laura Valencia,[‡] Aurora Rodríguez-Rodríguez,*[,†] Carlos Platas-Iglesias,*[,†] Peter Caravan,[§] and David Esteban-Gómez[†]

[†] Universidade da Coruña, Centro de Investigacións Científicas Avanzadas (CICA) and Departamento de Química, Facultade de Ciencias, 15071, A Coruña, Galicia, Spain

[‡] Departamento de Química Inorgánica, Facultad de Ciencias, Universidade de Vigo, As Lagoas, Marcosende, 36310 Pontevedra, Spain

[§] The Institute for Innovation in Imaging and the A. A. Martinos Center for Biomedical Imaging, Massachusetts General Hospital, Harvard Medical School, 149 13th Street, Suite 2301, Charlestown, Massachusetts 02129, United States

* Corresponding authors: aurora.rodriguez@udc.es (A. Rodríguez-Rodríguez); carlos.platas.iglesias@udc.es (C. Platas-Iglesias)

Contents

Table S1. Stability constants reported in the literature for the Mn(II) complexes, ligand protonation constants and pMn values. S3

Table S2. Crystal data and structure refinement details. S13

Figure S1. Structures of macrocyclic ligands based on 12-membered macrocycles L51-L85. ....S14

Figure S2. Structures of macrocyclic ligands based on 15-membered macrocycles L86-L105 and a H_4AAZTA derivative L106. ....S15

Figure S3. Structures of macrocyclic ligands based on 9-membered macrocycles L107-L119. ....S16

Figure S4. Structures of acyclic ligands L120-L145. ....S17

Figure S5. Structures of acyclic ligands L146-L168 and bispidine derivatives L169-L171. ....S18

References...S19

Table S1. Stability constants reported in the literature for the Mn(II) complexes, ligand protonation constants and pMn values.

	Ionic strength	Ligand protonation constants		Complex stability and protonation constants		pMn	Ref.
**L1**	0.1 M KNO_3	log K_1	10.55	log K_{MnL}	15.50	12.07	1
		log K_2	8.60				
		log K_3	4.26				
		log K_4	2.41				
		log K_5	2.08				
**L2**	0.1 M KCl	log K_1	9.50	log K_{MnL}	13.57	11.00	2
		log K_2	8.80	log K_{MnLH}	3.45		
		log K_3	2.90				
		log K_4	2.00				
**L3**	0.1 M KCl (ligands)	log K_1	10.29	log K_{MnL}	10.01	7.44	3
	0.1 M KNO_3 (complex)	log K_2	7.94				
		log K_3	2.70				
		log K_4	1.94				
**L4**	0.15 M NaCl	log K_1	9.17	log K_{MnL}	12.46	11.62	4
		log K_2	5.99	log K_{MnLH}	2.95		
		log K_3	2.73				
		log K_4	2.01				
		log K_5	1.38				
**L5**	0.15 M NaCl	log K_1	9.49	log K_{MnL}	10.78	9.44	5
		log K_2	7.17				
		log K_3	2.34				
**L6**	0.1 M KNO_3	log K_1	11.22	log K_{MnL}	14.10	11.20	6
		log K_2	6.29	log K_{MnLH}	3.46		
		log K_3	2.69				
		log K_4	1.76				
**L7**	0.1 M KNO_3	log K_1	9.70	log K_{MnL}	7.44	6.36	7
		log K_2	2.52				
		log K_3	1.70				
**L8**	0.15 NaCl	log K_1	8.44	log K_{MnL}	14.13	13.39	8
		log K_2	7.94	log K_{MnLH}	2.78		
		log K_3	2.76	log K_{MnLH2}	2.32		
		log K_4	1.91				
**L9**	0.1 M KCl	log K_1	10.33	log K_{MnL}	11.37	10.83	9
		log K_2	9.00	log K_{MnLH}	7.66		
		log K_3	5.86	log K_{MnLH2}	6.60	12.29	10
**L10**	0.15 M NaCl	log K_1	10.16	log K_{MnL}	14.14		
		log K_2	6.39	log K_{MnLH}	2.43		
		log K_3	3.13				
**L11**	0.1 M NaCl	log K_1	11.57	log K_{MnL}	15.10	10.34	11
		log K_2	11.16	log K_{MnLH}	9.35		
		log K_3	8.17	log K_{MnLH2}	8.55		
		log K_4	6.92	log K_{MnLH3}	6.41		
		log K_5	6.14	log K_{MnLH4}	5.76		
		log K_6	5.35				
		log K_7	3.34				
		log K_8	2.05				
**L12**	0.15 M NaCl	log K_1	10.36	log K_{MnL}	13.53	11.55	12
		log K_2	6.13	log K_{MnLH}	6.44		
		log K_3	3.68				
		log K_4	2.43				
**L13**	0.15 M NaCl	log K_1	7.26	log K_{MnL}	13.19	13.91	13
		log K_2	3.90	log K_{MnLH}	2.90		
		log K_3	3.29	log $K_{MnL(OH)}$	11.97		
		log K_4	1.77				
**L14**	0.1 M NaCl	log K_1	9.43	log K_{MnL}	14.69	13.59	14
		log K_2	6.01	log K_{MnLH}	2.42		
		log K_3	3.68				
		log K_4	2.51				
**L15**	0.15 M NaCl	log K_1	11.00	log K_{MnL}	14.19	11.54	15
		log K_2	5.20	log K_{MnLH}	2.85		
		log K_3	3.41				
		log K_4	2.30				

		log K_5	1.41				
**L16**	0.15 M NaCl	log K_1	6.66	log K_{MnL}	11.79	12.67	16
		log K_2	4.85	log K_{MnLH}	2.84		
		log K_3	3.53	log K_{MnLH2}	1.91		
		log K_4	3.32				
**L17**	0.1 M NaCl	log K_1	11.36	log K_{MnL}	14.16	9.21	14
		log K_2	9.85	log K_{MnLH}	7.75		
		log K_3	3.94				
		log K_4	3.40				
**L18**	0.1 M NaCl	log K_1	10.22	log K_{MnL}	13.66	11.06	14
		log K_2	8.05	log K_{MnLH}	4.49		
		log K_3	3.32				
		log K_4	2.43				
**L19**	0.1 M NaCl	log K_1	12.58	log K_{MnL}	14.61	8.41	14
		log K_2	9.87	log K_{MnLH}	7.73		
		log K_3	3.99				
		log K_4	2.97				
**L20**	0.15 M NaCl	log K_1	11.69	log K_{MnL}	14.64	8.95	17
		log K_2	9.75	log K_{MnLH}	4.40		
		log K_3	3.97				
		log K_4	2.68				
**L21**	0.1 M NaCl	log K_1	8.74	log K_{MnL}	13.88	13.09	18
		log K_2	7.58	log K_{MnLH}	2.77		
		log K_3	3.99				
		log K_4	2.39				
**L22**	0.15 M NaCl	log K_1	8.05	log K_{MnL}	9.38	9.32	19
		log K_2	7.43				
		log K_3	2.06				
**L23**	0.1 M $Me_4N(NO_3)$	log K_1	11.35	log K_{MnL}	16.74	11.41	20
		log K_2	9.73	log K_{MnLH}	3.91		
**L24**	0.1 M $Me_4N(NO_3)$	log K_1	10.52	log K_{MnL}	11.27	6.51	20
		log K_2	10.175				
		log K_3	4.085				
		log K_4	3.347				
**L25**	0.1 M KCl	log K_1	11.99	log K_{MnL}	19.43	13.68	21
		log K_2	9.51	log K_{MnLH}	3.37		
		log K_3	4.30				
		log K_4	3.63				
		log K_5	1.84				
**L26**	0.15 M NaCl	log K_1	12.55	log K_{MnL}	17.45	8.82	18
		log K_2	11.37	log K_{MnLH}	8.06		
		log K_3	8.57	log K_{MnLH2}	7.05		
		log K_4	7.02	log K_{MnLH3}	5.31		
		log K_5	5.36				
		log K_6	1.84				
**L27**	0.1 M KCl	log K_1	11.11	log K_{MnL}	11.54	7.91	22
		log K_2	8.22	log $K_{MnL(OH)}$	10.54		
**L28**	0.1 M NMe_4Cl	log K_1	11.22	log K_{MnL}	9.39	6.58	23
		log K_2	6.78	log $K_{MnL(OH)}$	11.13		
**L29**	0.15 M NaCl	log K_1	7.73	log K_{MnL}	13.03	10.99	24
		log K_2	7.66	log K_{MnLH}	2.40		
		log K_3	2.13	log $K_{MnL(OH)}$	11.49		
**L30**	0.1 M KCl	log K_1	9.62	log K_{MnL}	10.72	9.34	22
		log K_2	6.90	log $K_{MnL(OH)}$	9.44		
**L31**	0.1 M KCl	log K_1	10.14	log K_{MnL}	12.64	9.83	25
		log K_2	8.38				
**L32**	0.1 M KCl	log K_1	11.40	log K_{MnL}	15.22	9.99	17
		log K_2	9.58	log K_{MnLH}	4.15		
		log K_3	3.74				
		log K_4	1.65				
**L33**	0.1 M NMe_4Cl	log K_1	12.84	log K_{MnL}	15.41	7.41	23
		log K_2	11.20	log K_{MnLH}	8.10		
		log K_3	7.75	log K_{MnLH2}	5.24		
		log K_4	4.94				
		log K_5	1.40				
**L34**	0.15 M NaCl	log K_1	13.60	log K_{MnL}	18.98	8.64	18
		log K_2	12.23	log K_{MnLH}	8.09		

		log K_3	8.63	log K_{MnLH2}	7.79		
		log K_4	7.45	log K_{MnLH3}	6.74		
		log K_5	5.84	log K_{MnLH4}	5.02		
		log K_6	5.02				
**L35**	0.1 M KCl	log K_1	11.41	log K_{MnL}	19.44	13.95	17,21
		log K_2	9.83	log K_{MnLH}	3.96		
		log K_3	4.38	log K_{MnLH2}	3.70		
		log K_4	4.63				
		log K_5	1.92				
		log K_6	1.58				
**L36**	0.15 M NaCl	log K_1	7.31	log K_{MnL}	11.96	12.65	18
		log K_2	6.07				
**L37**	0.15 M NaCl	log K_1	9.97	log K_{MnL}	16.83	15.13	18
		log K_2	6.73	log K_{MnLH}	1.96		
		log K_3	3.22				
		log K_4	1.40				
**L38**	0.15 M NaCl	log K_1	10.72	log K_{MnL}	15.53	12.15	26
		log K_2	8.37	log K_{MnLH}	3.06		
		log K_3	3.81				
		log K_4	1.26				
**L39**	0.15 M NaCl	log K_1	12.25	log K_{MnL}	17.09	13.18	26
		log K_2	5.97	log K_{MnLH}	2.14		
		log K_3	3.47				
		log K_4	1.99				
**L40**	0.1 M NMe_4Cl	log K_1	12.32	log K_{MnL}	10.61	6.35	27
		log K_2	7.89	log K_{MnLH}	6.32		
		log K_3	5.44	log $K_{MnL(OH)}$	-1.81		
		log K_4	1.88				
**L41**	0.1 M NMe_4Cl	log K_1	8.42	log K_{MnL}	4.30	*	27
		log K_2	1.39	log $K_{MnL(OH)}$	-6.30		
**L42**	1 M KCl	log K_1	11.82	log K_{MnL}	11.56	8.02	28
		log K_2	6.70				
		log K_3	2.87				
		log K_4	1.02				
**L43**	0.1 M KNO_3	log K_1	10.57	log K_{MnL}	7.73	6.13	29
		log K_2	4.02				
		log K_3	1.80				
**L44**	0.15 M NaCl	log K_1	9.97	log K_{MnL}	14.19	12.52	30
		log K_2	6.42	log K_{MnLH}	2.61		
		log K_3	3.75				
		log K_4	1.94				
**L45**	0.15 M NaCl	log K_1	10.24	log K_{MnL}	11.00	9.10	30
		log K_2	5.78	log K_{MnLH}	3.36		
		log K_3	3.67				
		log K_4	2.18				
**L46**	0.15 M NaCl	log K_1	10.29	log K_{MnL}	10.67	8.72	30
		log K_2	5.90	log K_{MnLH}	4.34		
		log K_3	3.96				
		log K_4	2.52				
**L47**	0.1 M $NaClO_4$	log K_1	10.31	log K_{MnL}	10.85	7.03	31
		log K_2	9.29	log K_{MnLH}	5.04		
		log K_3	5.93	log $K_{MnL(OH)}$	11.22		
**L48**	0.1 M $NaClO_4$	log K_1	10.61	log K_{MnL}	11.09	6.92	31
		log K_2	9.37	log K_{MnLH}	4.99		
		log K_3	5.54	log $K_{MnL(OH)}$	11.14		
**L49**	0.1 M Me_4NCl	log K_1	9.40	log K_{MnL}	10.89	8.67	32
		log K_2	8.54	log K_{MnLH}	4.27		
		log K_3	5.28	log $K_{MnL(OH)}$	11.52		
**L50**	0.1 M Me_4NCl	log K_1	8.82	log K_{MnL}	7.18	6.40	32
		log K_2	7.80	log $K_{MnL(OH)}$	11.69		
**L51**	0.1 M KNO_3	log K_1	10.33	log K_{MnL}	8.81	6.47	33
		log K_2	7.83				
		log K_3	1.27				
**L52**	0.15 M NaCl	log K_1	11.44	log K_{MnL}	15.68	10.48	17
		log K_2	9.51	log K_{MnLH}	4.15		
		log K_3	4.14				
		log K_4	1.55				

**L53**	0.1 M Me_4NCl	$\log K_1$	11.07	$\log K_{MnL}$	16.13	11.05	34
		$\log K_2$	9.76	$\log K_{MnLH2}$	8.31		
		$\log K_3$	3.84	$\log K_{MnL(OH)}$	2.49		
		$\log K_4$	1.75				
**L54**	0.1 M Me_4NCl	$\log K_1$	12.46	$\log K_{MnL}$	19.40	13.20	34,35
		$\log K_2$	9.49	$\log K_{MnLH}$	3.13		
		$\log K_3$	4.26				
		$\log K_4$	3.51				
		$\log K_5$	1.97				
**L55**	0.1 M KCl (230 °C)	--		$\log K_{MnL}$	18.8	-*	36
**L56**	0.1 M KCl	$\log K_1$	12.34	$\log K_{MnL}$	20.1	13.57	21
		$\log K_2$	11.02	$\log K_{MnLH}$	10.30		
		$\log K_3$	9.22	$\log K_{MnLH2}$	3.56		
		$\log K_4$	4.43	$\log K_{MnLH3}$	2.93		
		$\log K_5$	2.60				
		$\log K_6$	1.52				
**L57**	0.1 M Me_4NCl	$\log K_1$	11.74	$\log K_{MnL}$	19.89	14.14	34,35
		$\log K_2$	9.76	$\log K_{MnLH}$	4.26		
		$\log K_3$	4.68	$\log K_{MnLH2}$	2.99		
		$\log K_4$	4.11				
		$\log K_5$	2.37				
**L58**	0.1 M $Me_4(NO_3)$	$\log K_1$	12.09	$\log K_{MnL}$	20.20	14.10	20
		$\log K_2$	9.763	$\log K_{MnLH}$	4.149		
		$\log K_3$	4.556				
		$\log K_4$	4.09				
**L59**	0.1 M NaCl	$\log K_1$	11.72	$\log K_{MnL}$	18.37	13.33	18
		$\log K_2$	9.06	$\log K_{MnLH}$	4.56		
		$\log K_3$	4.74				
		$\log K_4$	5.59				
		$\log K_5$	1.96				
**L60**	0.1 M Me_4NCl	$\log K_1$	11.17	$\log K_{MnL}$	17.89	13.14	34,35
		$\log K_2$	9.33	$\log K_{MnLH}$	5.07		
		$\log K_3$	4.99				
		$\log K_4$	3.80				
		$\log K_5$	2.84				
**L61**	0.1 M KNO_3	$\log K_1$	9.74	$\log K_{MnL}$	3.70	-*	33
		$\log K_2$	8.67	$\log K_{MnL(OH)}$	9.10		
		$\log K_3$	4.67				
**L62**	0.15 M NaCl	$\log K_1$	11.34	$\log K_{MnL}$	19.01	14.48	37
		$\log K_2$	8.93	$\log K_{MnLH}$	6.88		
		$\log K_3$	6.91	$\log K_{MnLH2}$	2.50		
		$\log K_4$	1.97				
**L63**	0.15 M NaCl	$\log K_1$	8.85	$\log K_{MnL}$	13.20	12.69	18
		$\log K_2$	4.55	$\log K_{MnLH}$	3.98		
		$\log K_3$	3.81	$\log K_{MnLH2}$	3.14		
		$\log K_4$	3.21	$\log K_{MnLH3}$	2.87		
		$\log K_5$	2.80	$\log K_{MnL(OH)}$	10.67		
		$\log K_6$	1.38				
**L64**	0.15 M NaCl	$\log K_1$	8.76	$\log K_{MnL}$	11.78	11.35	18
		$\log K_2$	4.10				
**L65**	0.15 M NaCl	$\log K_1$	8.74	$\log K_{MnL}$	14.05	13.64	18
		$\log K_2$	5.77				
		$\log K_3$	1.42				
**L66**	0.1 M Me_4NCl	$\log K_1$	10.47	$\log K_{MnL}$	11.54	8.10	38
		$\log K_2$	8.71	$\log K_{MnLH}$	4.95		
		$\log K_3$	2.79				
**L67**	0.1 M Me_4NCl	$\log K_1$	11.84	$\log K_{MnL}$	14.06	8.31	38
		$\log K_2$	9.64	$\log K_{MnLH}$	5.35		
		$\log K_3$	6.23	$\log K_{MnL(OH)}$	11.97		
		$\log K_4$	0.99				
**L68**	0.1 M $Me_4(NO_3)$	$\log K_1$	10.90	$\log K_{MnL}$	18.59	15.89	39
		$\log K_2$	7.11	$\log K_{MnLH}$	2.21		
		$\log K_3$	3.88	$\log K_{MnL(OH)}$	8.71		
		$\log K_4$	2.27				
**L69**	0.1 M KNO_3	$\log K_1$	9.79	$\log K_{MnL}$	7.29	-*	33
		$\log K_2$	8.49	$\log K_{MnL(OH)}$	9.93		

		log K_3	2.85				
**L70**	0.1 M KNO_3	log K_1	9.92	log K_{MnL}	5.477	-*	33
		log K_2	8.56				
		log K_3	4.66				
**L71**	0.1 M Me_4NCl	log K_1	11.29	log K_{MnL}	14.66	9.28	40
		log K_2	9.84	log K_{MnLH}	4.60		
		log K_3	3.97	log K_{MnLH2}	4.01		
		log K_4	2.59				
**L72**	0.1 M KCl	log K_1	11.66	log K_{MnL}	15.07	9.41	17
		log K_2	9.75	log K_{MnLH}	4.48		
		log K_3	4.06				
		log K_4	2.58				
**L73**	0.1 M Me_4NCl	log K_1	11.29	log K_{MnL}	14.54	9.16	34
		log K_2	9.84	log K_{MnLH}	4.25		
		log K_3	3.97	log K_{MnLH2}	4.45		
		log K_4	2.59	log $K_{MnL(OH)}$	2.50		
**L74**	0.15 M NaCl	log K_1	7.19	log K_{MnL}	7.76	8.49	19
		log K_2	6.29				
**L75**	0.15 M NaCl	log K_1	10.65	log K_{MnL}	15.87	13.53	41
		log K_2	6.55	log K_{MnLH}	6.03		
		log K_3	5.84	log K_{MnLH2}	4.14		
		log K_4	4.39				
**L76**	0.15 M NaCl	log K_1	10.5	log K_{MnL}	14.86	12.64	42
		log K_2	6.93				
		log K_3	2.36				
		log K_4	1.64				
**L77**	0.1 M KNO_3	log β_2	26.41	log K_{MnL}	12.35	-*	43
		log K_3	6.78	log K_{MnLH}	8.87		
		log K_4	1.64				
**L78**	0.15 M NaCl	log K_1	11.22	log K_{MnL}	14.48	9.63	44
		log K_2	9.38	log K_{MnLH}	4.03		
		log K_3	3.39				
**L79**	0.1 M Me_4NNO_3	log K_1	11.24	log K_{MnL}	12.737	9.83	45
		log K_2	6.02	log K_{MnLH}	3.143		
		log K_3	2.94				
		log K_4	1.38				
**L80**	0.1 M Me_4NNO_3	log K_1	10.54	log K_{MnL}	10.72	8.53	45
		log K_2	4.84				
		log K_3	1.00				
**L81**	0.1 M Me_4NNO_3	log K_1	9.532	log K_{MnL}	11.03	9.52	45
		log K_2	7.46				
		log K_3	2.11				
**L82**	0.1 M Me_4NNO_3	log K_1	11.03	log K_{MnL}	7.08	-*	46
		log K_2	6.97	log K_{MnLH}	6.87		
		log K_3	3.58				
		log K_4	0.80				
**L83**	0.1 M Me_4NNO_3	log K_1	10.25	log K_{MnL}	9.18	6.49	46
		log K_2	8.33	log $K_{MnL(OH)}$	10.63		
		log K_3	5.52				
		log K_4	2.25				
		log K_5	1.30				
**L84**	0.1 M Me_4NNO_3	log K_1	11.06	log K_{MnL}	9.99	7.13	46
		log K_2	7.15				
		log K_3	3.63				
**L85**	0.1 M Me_4NNO_3	log K_1	10.27	log K_{MnL}	11.81	9.27	46
		log K_2	7.90				
		log K_3	5.18				
		log K_4	2.40				
**L86**	0.1 M $NaClO_4$	log K_1	10.38	log K_{MnL}	10.55	6.55	47
		log K_2	9.51	log K_{MnLH}	3.66		
		log K_3	5.99				
**L87**	0.1 M $NaClO_4$	log K_1	10.64	log K_{MnL}	11.65	7.36	31
		log K_2	9.42	log K_{MnLH}	4.90		
		log K_3	5.60	log $K_{MnL(OH)}$	11.35		
**L88**	0.1 M $NaClO_4$	log K_1	10.61	log K_{MnL}	11.09	6.92	31
		log K_2	9.37	log K_{MnLH}	4.99		
		log K_3	5.54	log $K_{MnL(OH)}$	11.14		

**L89**	0.1 M $NaClO_4$	log K_1	10.72	log K_{MnL}	10.74	6.50	31
		log K_2	9.45	log K_{MnLH}	5.80		
		log K_3	5.61	log $K_{MnL(OH)}$	11.36		
**L90**	0.1 M $NaClO_4$	log K_1	10.12	log K_{MnL}	10.19	6.72	31
		log K_2	9.18	log K_{MnLH}	5.34		
		log K_3	5.97	log $K_{MnL(OH)}$	11.35		
**L91**	0.1 M $NaClO_4$	log K_1	10.54	log K_{MnL}	11.02	6.96	31
		log K_2	9.32	log K_{MnLH}	5.33		
		log K_3	5.89	log $K_{MnL(OH)}$	11.11		
**L92**	0.1 M $NaClO_4$	log K_1	10.72	log K_{MnL}	11.48	7.31	31
		log K_2	9.22	log K_{MnLH}	5.36		
		log K_3	5.74	log $K_{MnL(OH)}$	11.23		
**L93**	0.1 M $NaClO_4$	log K_1	9.96	log K_{MnL}	10.19	6.88	31
		log K_2	9.16	log K_{MnLH}	5.83		
		log K_3	5.80	log $K_{MnL(OH)}$	10.77		
**L94**	0.1 M $NaClO_4$	log K_1	10.33	log K_{MnL}	10.93	7.25	31
		log K_2	9.12	log K_{MnLH}	5.21		
		log K_3	5.67	log $K_{MnL(OH)}$	11.23		
**L95**	0.1 M $NaClO_4$	log K_1	11.56	log K_{MnL}	11.97	6.83	31
		log K_2	9.41	log K_{MnLH}	5.82		
		log K_3	5.61	log $K_{MnL(OH)}$	11.32		
**L96**	0.1 M $NaClO_4$	log K_1	9.89	log K_{MnL}	10.46	7.18	31
		log K_2	9.17	log K_{MnLH}	5.60		
		log K_3	5.80	log $K_{MnL(OH)}$	10.88		
**L97**	0.1 M $NaClO_4$	log K_1	10.36	log K_{MnL}	10.88	7.05	31
		log K_2	9.26	log K_{MnLH}	5.28		
		log K_3	5.79	log $K_{MnL(OH)}$	11.27		
**L98**	0.1 M $NaClO_4$	log K_1	9.43	log K_{MnL}	11.64	9.14	31
		log K_2	8.80	log K_{MnLH}	4.20		
		log K_3	5.28	log $K_{MnL(OH)}$	11.54		
**L99**	0.1 M $NaClO_4$	log K_1	9.11	log K_{MnL}	11.12	8.93	47
		log K_2	8.82	log K_{MnLH}	4.51		
		log K_3	5.27				
**L100**	0.1 M $NaClO_4$	log K_1	9.85	log K_{MnL}	11.46	8.44	47
		log K_2	8.91	log K_{MnLH}	4.11		
		log K_3	5.15				
**L101**	0.1 M $NaClO_4$	log K_1	9.96	log K_{MnL}	10.96	7.42	48
		log K_2	9.35	log K_{MnLH}	5.66		
		log K_3	5.24				
**L102**	0.1 M KNO_3	log K_1	9.51	log K_{MnL}	6.63	-*	49
		log K_2	8.47				
		log K_3	2.30				
**L103**	0.15 M NaCl	log K_1	8.53	log K_{MnL}	5.62	6.05	49
		log K_2	7.63	log $K_{MnL(OH)}$	10.50		
**L104**	0.1 M Me_4NNO_3	log K_1	9.067	log K_{MnL}	12.11	10.22	50
		log K_2	8.544				
		log K_3	1.75				
**L105**	0.1 M Me_4NNO_3	log K_1	10.02	log K_{MnL}	14.44	12.13	50
		log K_2	7.93	log K_{MnLH}	3.98		
		log K_3	3.93				
		log K_4	2.41				
**L106**	0.15 M NaCl	log K_1	10.90	log K_{MnL}	11.43	8.88	30
		log K_2	5.14	log K_{MnLH}	3.36		
		log K_3	3.71				
		log K_4	2.17				
**L107**	0.1 M KNO_3	log K_1	9.677	log K_{MnL}	3.00	-*	29
		log K_2	5.45				
**L108**	0.1 M Me_4NCl	log K_1	9.23	log K_{MnL}	4.82	-*	27
		log K_2	1.85	log $K_{MnL(OH)}$	-6.02		
**L109**	0.1 M KNO_3	log K_1	10.42	log K_{MnL}	5.80	-*	51
		log K_2	6.82				
**L110**	0.1 M Me_4NCl	log K_1	10.59	log K_{MnL}	7.43	-*	27
		log K_2	3.99	log $K_{MnL(OH)}$	-3.42		
		log K_3	1.83				
**L111**	0.1 M KCl	log K_1	11.20	log K_{MnL}	9.90	7.09	52
		log K_2	5.35				
		log K_3	4.07				

		$\log K_4$	3.04				
		$\log K_5$	2.00				
**L112**	0.1 M KCl	$\log K_1$	11.10	$\log K_{MnL}$	10.15	7.41	52
		$\log K_2$	6.00	$\log K_{MnLH}$	4.95		
		$\log K_3$	4.53	$\log K_{MnL(OH)}$	9.65		
		$\log K_4$	2.74				
**L113**	0.1 M KCl	$\log K_1$	12.00	$\log K_{MnL}$	10.98	7.35	52
		$\log K_2$	5.81				
		$\log K_3$	2.71				
**L114**	0.1 M Me_4NCl	$\log K_1$	11.41	$\log K_{MnL}$	14.90	11.83	53,54
		$\log K_2$	5.74				
		$\log K_3$	3.16				
		$\log K_4$	1.71				
**L115**	0.1 M KNO_3	$\log K_1$	11.79	$\log K_{MnL}$	16.60	11.72	55
		$\log K_2$	8.65	$\log K_{MnLH}$	10.80		
		$\log K_3$	7.09	$\log K_{MnLH2}$	7.30		
		$\log K_4$	5.38	$\log K_{MnLH3}$	4.40		
		$\log K_5$	2.53				
**L116**	0.15 M NaCl	$\log K_1$	11.26	$\log K_{MnL}$	15.29	9.20	12
		$\log K_2$	10.59	$\log K_{MnLH}$	5.52		
		$\log K_3$	4.57				
		$\log K_4$	2.69				
**L117**	0.15 M NaCl	$\log K_1$	12.05	$\log K_{MnL}$	15.60	11.89	12
		$\log K_2$	5.77				
		$\log K_3$	3.30				
		$\log K_4$	2.20				
**L118**	0.15 M NaCl	$\log K_1$	11.33	$\log K_{MnL}$	10.28	7.07	44
		$\log K_2$	7.30	$\log K_{MnL(OH)}$	11.94		
		$\log K_3$	2.49				
**L119**	0.1 M NaCl	$\log K_1$	11.41	$\log K_{MnL}$	10.56	7.20	56
		$\log K_2$	7.46	$\log K_{MnL(OH)}$	11.62		
		$\log K_3$	3.25				
**L120**	Not employed	--		$\log K_{MnL}$	4.40	-	57
**L121**	0.15 M NaCl	$\log K_1$	8.99	$\log K_{MnL}$	13.80	13.14	58
		$\log K_2$	5.75	$\log K_{MnLOH}$	2.56		
		$\log K_3$	3.67				
		$\log K_4$	2.47				
		$\log K_5$	1.37				
**L122**	0.1 M KCl	$\log K_1$	10.58	$\log K_{MnL}$	15.60	12.15	59
		$\log K_2$	8.60				
		$\log K_3$	4.27				
		$\log K_4$	2.64				
		$\log K_5$	1.50				
**L123**	1 M $NaClO_4$	$\log K_1$	8.85	$\log K_{MnL}$	14.04	13.50	60
		$\log K_2$	6.28				
		$\log K_3$	2.30				
		$\log K_4$	2.20				
		$\log K_5$	1.40				
**L124**	0.2 M $NaClO_4$	$\log K_1$	10.26	$\log K_{MnL}$	13.64	11.71	61
		$\log K_2$	6.16				
		$\log K_3$	2.67				
		$\log K_4$	2.00				
**L125**	0.1 M KCl	$\log K_1$	10.26	$\log K_{MnL}$	13.50	11.58	36
		$\log K_2$	6.01				
		$\log K_3$	2.84				
		$\log K_4$	2.39				
**L126**	0.1 M KCl	$\log K_1$	10.04	$\log K_{MnL}$	13.80	12.09	36
		$\log K_2$	6.18				
		$\log K_3$	3.01				
		$\log K_4$	2.36				
**L127**	1 M $NaClO_4$	$\log K_1$	6.41	$\log K_{MnL}$	11.37	12.28	62
		$\log K_2$	4.61	$\log K_{MnLH}$	2.29		
		$\log K_3$	3.53	$\log K_{MnLH2}$	1.70		
		$\log K_4$	3.00				
**L128**	0.15 M NaCl	$\log K_1$	9.36	$\log K_{MnL}$	14.32	13.29	4
		$\log K_2$	5.95	$\log K_{MnLH}$	2.90		
		$\log K_3$	3.62				

		log K_4	2.57				
		log K_5	1.49				
**L129**	0.15 M NaCl	log K_1	8.83	log K_{MnL}	10.63	10.11	63
		log K_2	6.22	log K_{MnLH}	3.42		
		log K_3	3.27				
		log K_4	2.03				
**L130**	0.5 M KCl	log K_1	9.888	log K_{MnL}	3.63	-*	64
		log K_2	6.972				
		log K_3	1.490				
**L131**	0.1 M KNO_3	log K_1	4.50	log K_{MnL}	0.81	-*	65
		log K_2	4.05				
		log K_3	3.33				
**L132**	0.1 M KNO_3	log K_1	12.46	log K_{MnL}	14.78	6.70	66
		log K_2	11.00	log K_{MnLH}	7.66		
		log K_3	8.32	log K_{MnLH2}	6.58		
		log K_4	4.64				
**L133**	0.1 M NaCl	log K_1	11.05	log K_{MnL}	13.07	11.01	14
		log K_2	8.83	log K_{MnLH}	7.29		
		log K_3	4.81				
		log K_4	2.22				
**L134**	0.1 M NaCl	log K_1	9.32	log K_{MnL}	11.29	9.98	14
		log K_2	7.48	log K_{MnLH}	4.96		
		log K_3	4.26				
		log K_4	2.67				
**L135**	0.1 M NaCl	log K_1	11.61	log K_{MnL}	13.32	8.77	14
		log K_2	9.10	log K_{MnLH}	7.61		
		log K_3	4.86				
		log K_4	2.46				
**L136**	0.1 M KCl	log K_1	9.73	log K_{MnL}	10.70	9.31	67
		log K_2	5.33				
		log K_3	2.64				
**L137**	0.15 M NaCl	log K_1	5.48	log K_{MnL}	9.55	10.50	13
		log K_2	4.51	log K_{MnLH}	4.84		
		log K_3	4.28	log K_{MnLH2}	2.51		
		log K_4	2.70				
**L138**	0.1 M KNO_3	--		log K_{MnL}	4.72	-	68
**L139**	0.1 M KNO_3	log K_1	10.79	log K_{MnL}	6.26	-*	68
		log K_2	6.08				
		log K_3	5.04				
		log K_4	0.86				
**L140**	0.1 M $KClO_4$	log K_1	9.80	log K_{MnL}	4.61	-*	69
		log K_2	5.04				
**L141**	0.15 M NaCl	log K_1	8.058	log K_{MnL}	9.59	9.80	70
		log K_2	3.462	log K_{MnLH}	2.90		
		log K_3	2.34				
		log K_4	1.79				
**L142**	0.1 M KCl	log K_1	9.68	log K_{MnL}	6.55	-*	71
		log K_2	9.10				
		log K_3	8.08				
		log K_4	4.72				
		log K_5	2.98				
**L143**	0.1 M KNO_3	log K_1	9.92	log K_{MnL}	4.90	-*	72
		log K_2	9.20				
		log K_3	6.67				
		log K_4	3.32				
**L144**	0.1 M KNO_3	log K_1	8.22	log K_{MnL}	7.10	7.20	73
		log K_2	2.70	log K_{MnLH}	3.50		
		log K_3	2.70				
**L145**	0.1 M NaCl	log K_1	10.75	log K_{MnL}	12.56	9.01	74
		log K_2	10.33	log K_{MnLH}	8.74		
		log K_3	7.12	log K_{MnLH2}	7.90		
		log K_4	5.64				
		log K_5	3.12				
		log K_6	1.74				
**L146**	0.15 M NaCl	log K_1	8.47	log K_{MnL}	13.89	13.23	8
		log K_2	7.79	log K_{MnLH}	3.03		
		log K_3	2.73				

		log K_4	2.77				
		log K_5	1.87				
**L147**	0.1 M KCl	log K_1	9.66	log K_{MnL}	10.26	8.94	75
		log K_2	5.84	log K_{MnLH}	4.07		
		log K_3	3.06				
		log K_4	2.08				
		log K_5	1.71				
**L148**	0.1 M KCl	log K_1	9.58	log K_{MnL}	14.71	13.46	75
		log K_2	6.00	log K_{MnLH}	3.25		
		log K_3	3.78				
		log K_4	2.32				
		log K_5	2.07				
**L149**	0.1 M KCl	log K_1	8.89	log K_{MnL}	15.81	15.20	75
		log K_2	6.61	log K_{MnLH}	3.60		
		log K_3	4.26				
		log K_4	2.97				
		log K_5	2.79				
**L150**	0.15 M NaCl	log K_1	8.08	log K_{MnL}	8.80	8.98	5
		log K_2	5.92				
		log K_3	2.61				
**L151**	0.1 M KCl (30 °C)	log K_1	8.78	log K_{MnL}	5.65	-*	76
		log K_2	1.96				
**L152**	0.1 M KCl	log K_1	8.96	log K_{MnL}	5.53	-*	77
		log K_2	2.20				
**L153**	0.1 M KCl	log K_1	11.05	log K_{MnL}	7.71	6.05	77
		log K_2	5.58				
**L154**	0.1 M $NaNO_3$	log K_1	5.23	log K_{MnL}	3.57	-*	78
				log K_{MnLH}	2.75		
**L155**	0.1 M $NaNO_3$	log K_1	8.79	log K_{MnL}	2.66	-*	79
		log K_2	2.04				
**L156**	0.1 M KNO_3	log K_1	8.23	log K_{MnL}	5.90	6.29	72
		log K_2	5.45				
		log K_3	1.81				
		log K_4	1.62				
**L157**	0.1 M	log K_1	7.30	log K_{MnL}	3.52	-*	80
		log K_2	2.60				
		log K_3	1.12				
**L158**	0.1 M	log K_1	6.17	log K_{MnL}	5.54	6.61	80
		log K_2	4.35				
		log K_3	2.55				
**L159**	0.1 M	log K_1	7.19	log K_{MnL}	10.13	10.88	80
		log K_2	4.85				
		log K_3	3.32				
		log K_4	2.85				
**L160**	0.1 M KNO_3	log K_1	10.74	log K_{MnL}	9.80	7.89	7
		log K_2	5.62	log K_{MnLH}	5.89		
		log K_3	2.31	log K_{MnLH2}	4.60		
		log K_4	1.70	log $K_{MnL(OH)}$	2.70		
**L161**	0.1 M KNO_3	log K_1	11.80	log K_{MnL}	9.90	6.87	7
		log K_2	6.41	log K_{MnLH}	6.73		
		log K_3	5.02	log K_{MnLH2}	4.77		
		log K_4	2.07	log K_{MnLH3}	3.10		
		log K_5	1.10	log $K_{MnL(OH)}$	2.60		
**L162**	0.1 M KNO_3	log K_1	12.80	log K_{MnL}	10.90	6.67	7
		log K_2	7.15	log K_{MnLH}	7.37		
		log K_3	5.89	log K_{MnLH2}	5.93		
		log K_4	4.63	log K_{MnLH3}	4.70		
		log K_5	1.40				
**L163**	0.15 M NaCl	log K_1	7.18	log K_{MnL}	3.34	-*	81
		log K_2	2.66				
**L164**	0.15 M NaCl	log K_1	6.16	log K_{MnL}	5.48	6.56	81
		log K_2	4.42				
**L165**	0.1 M KCl	log K_1	9.43	log K_{MnL}	12.28	9.77	2
		log K_2	8.82	log K_{MnLH}	4.43		
		log K_3	2.77				
		log K_4	2.06				
		log K_5	1.88				

**L166**	0.15 M NaCl	log K_1	11.67	log K_{MnL}	11.20	7.89	82
		log K_2	5.47	log K_{MnLH}	3.77		
		log K_3	2.74				
		log K_4	1.62				
**L167**	0.15 M NaCl	log K_1	12.40	log K_{MnL}	13.69	7.05	82
		log K_2	10.14	log K_{MnLH}	6.80		
		log K_3	4.76	log K_{MnLH2}	5.60		
		log K_4	1.91				
**L168**	0.1 M KCl	log K_1	10.28	log K_{MnL}	10.55	8.57	3
		log K_2	6.52				
		log K_3	2.66				
		log K_4	1.93				
**L169**	0.15 M NaCl	log K_1	11.05	log K_{MnL}	24.20	21.42	83
		log K_2	6.73	log K_{MnLH}	3.04		
		log K_3	5.62				
		log K_4	5.27				
		log K_5	2.30				
**L170**	0.15 M NaCl	log K_1	11.90	log K_{MnL}	24.70	21.15	83
		log K_2	5.44				
		log K_3	5.28				
		log K_4	1.36				
**L171**	0.1 M KCl	log K_1	11.44	log K_{MnL}	12.21	9.23	84
		log K_2	10.31	log K_{MnLH}	10.42		
		log K_3	4.71	log K_{MnLH2}	3.87		
		log K_4	2.76				
		log K_5	2.22				

* Excluded from the fit because the complex is nearly fully dissociated under the conditions used to define pMn.

Table S2. Crystal data and structure refinement details.

**Parameter**	**Value**
Formula	$C_{16}H_{28}Mn_2N_4O_{10}$
Molecular weight, MW	546.30
Crystal system	Tetragonal
Space group	P4/m
a	12.7533(3) Å
b	12.7533(3) Å
c	17.8525(9) Å
V	2903.6(2) Å^3
F(000)	1128
Z	4
D_{calc}	1. 250 g cm^{-3}
μ	0.915
θ range	2.79°-28.33°
R_{int}	0.0365
Measured reflections	25435[a]
Goodness of fit, GOF on F^2	1.074
R1	0.0499
wR2 (all data)	0.1222
Larg. Diff. peak and hole (eÅ^{-3})	0.986 and 0.430

[a] Of which 3706 were independent and 3261 were unique, with I >2σ (I).

Crystal structure determinations. A suitable crystal of **$[Mn_2(DOTA)(H_2O)_2]$** was analysed by X-ray diffraction at 100 K using a **Bruker D8 Venture diffractometer with a Photon 100 CMOS detector and Mo-Kα radiation** (λ = 0.71073 Å) generated by an Incoatec high brillance microfocus source equipped with Incoatec Helios multilayer optics. The software APEX3[85] was used for collecting frames of data, indexing reflections, and the determination of lattice parameters, SAINT[86] for integration of intensity of reflections, and SADABS[87] for scaling and empirical absorption correction. The structure was solved by dual-space methods using the program SHELXT.[88] All non-hydrogen atoms were refined with anisotropic thermal parameters by full-matrix least-squares calculations on F^2 using the program SHELXL-2014.[89] Hydrogen atoms positions were calculated and constrained with isotropic thermal parameters except for the hydrogen atom of the water molecules, which were located from a Fourier-difference map and refined isotropically. Highly disordered solvent molecules were removed using the Solvent Mask routine from OLEX 2.[90] CCDC 2184025 contains the supplementary crystallographic data for **$[Mn_2(DOTA)(H_2O)_2]$**.These data can be obtained free of charge from the Cambridge Crystallographic Data Centre via www.ccdc.ac.uk/data_request/cif. A summary of the crystallographic data and the structure refinement parameters is reported in Table S2.

Figure S1. Structures of macrocyclic ligands based on 12-membered macrocycles **L51-L85**.

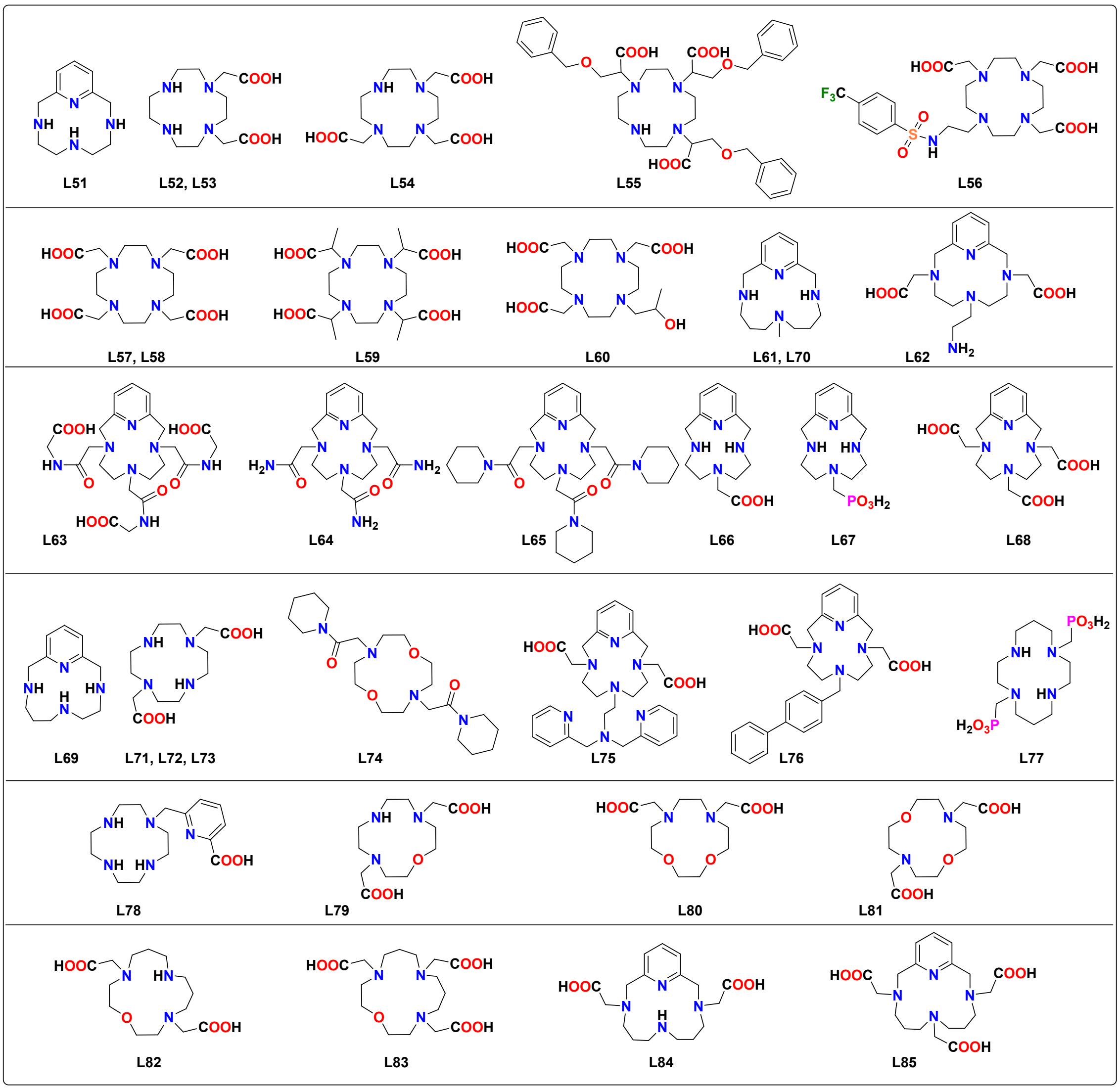

Figure S2. Structures of macrocyclic ligands based on 15-membered macrocycles **L86-L105** and a H_4AAZTA derivative **L106**.

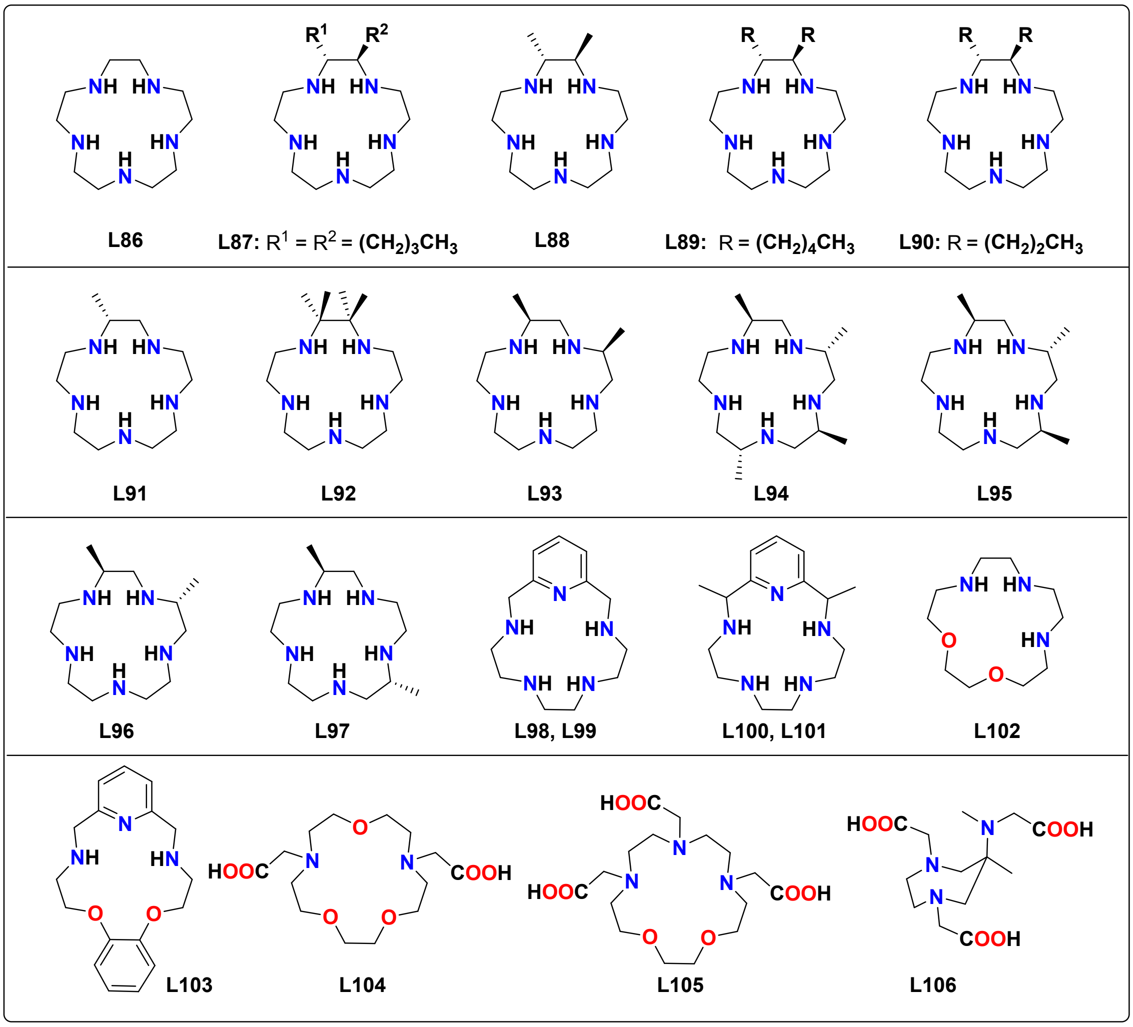

Figure S3. Structures of macrocyclic ligands based on 9-membered macrocycles **L107-L119**.

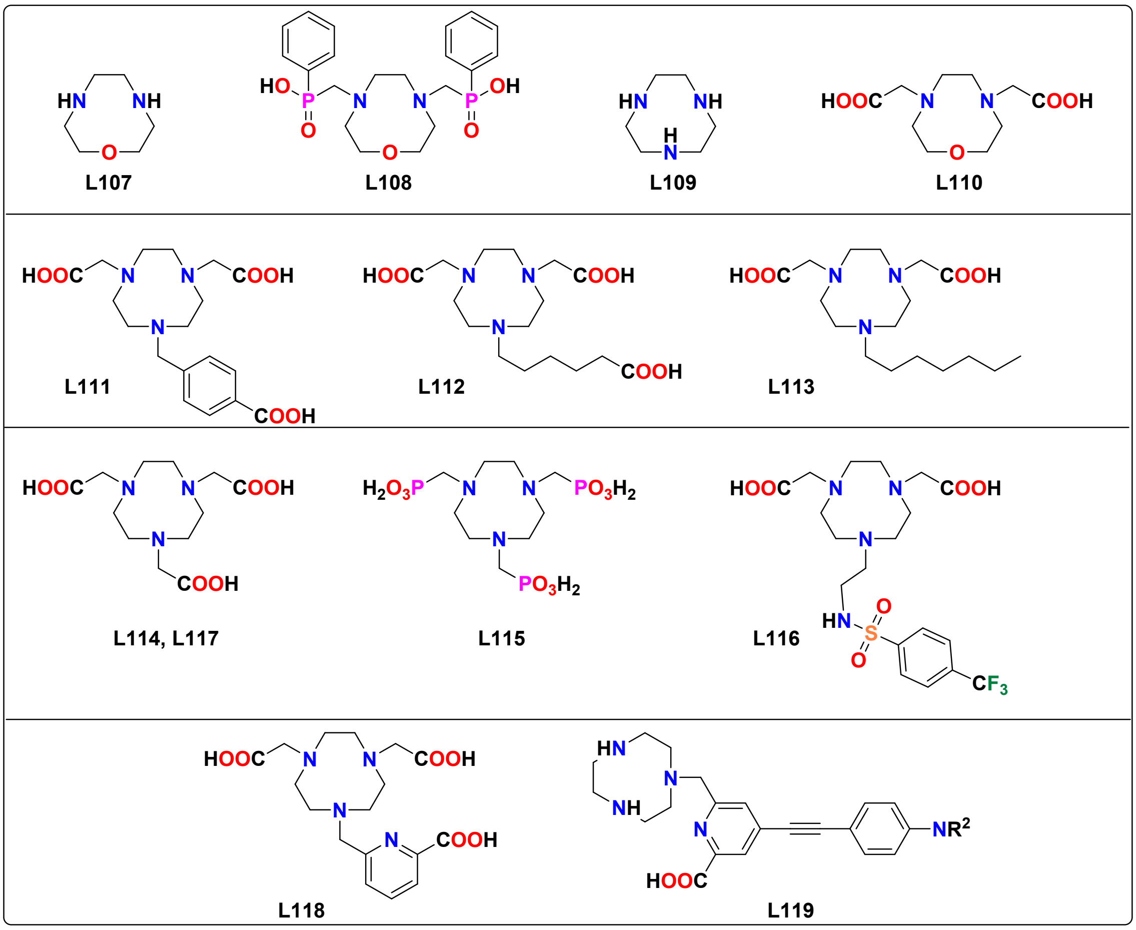

Figure S4. Structures of acyclic ligands **L120-L145**.

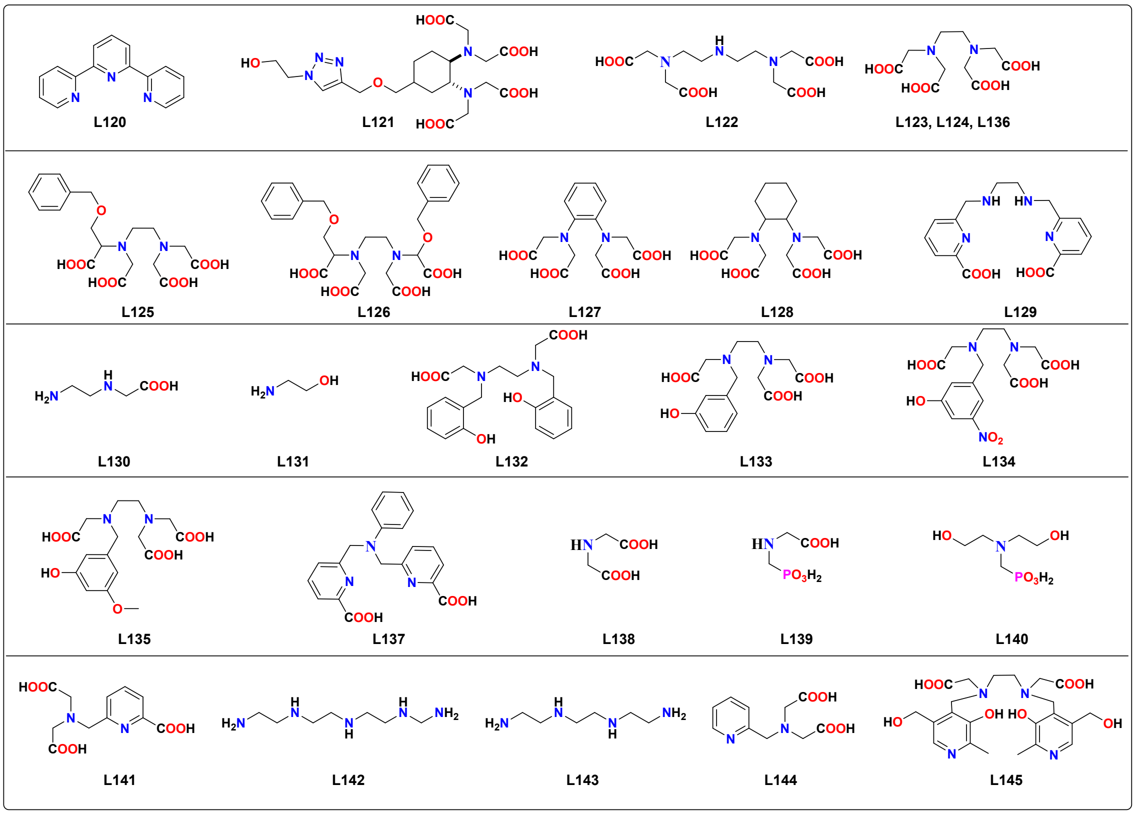

Figure S5. Structures of acyclic ligands **L146-L168** and bispidine derivatives **L169-L171**.

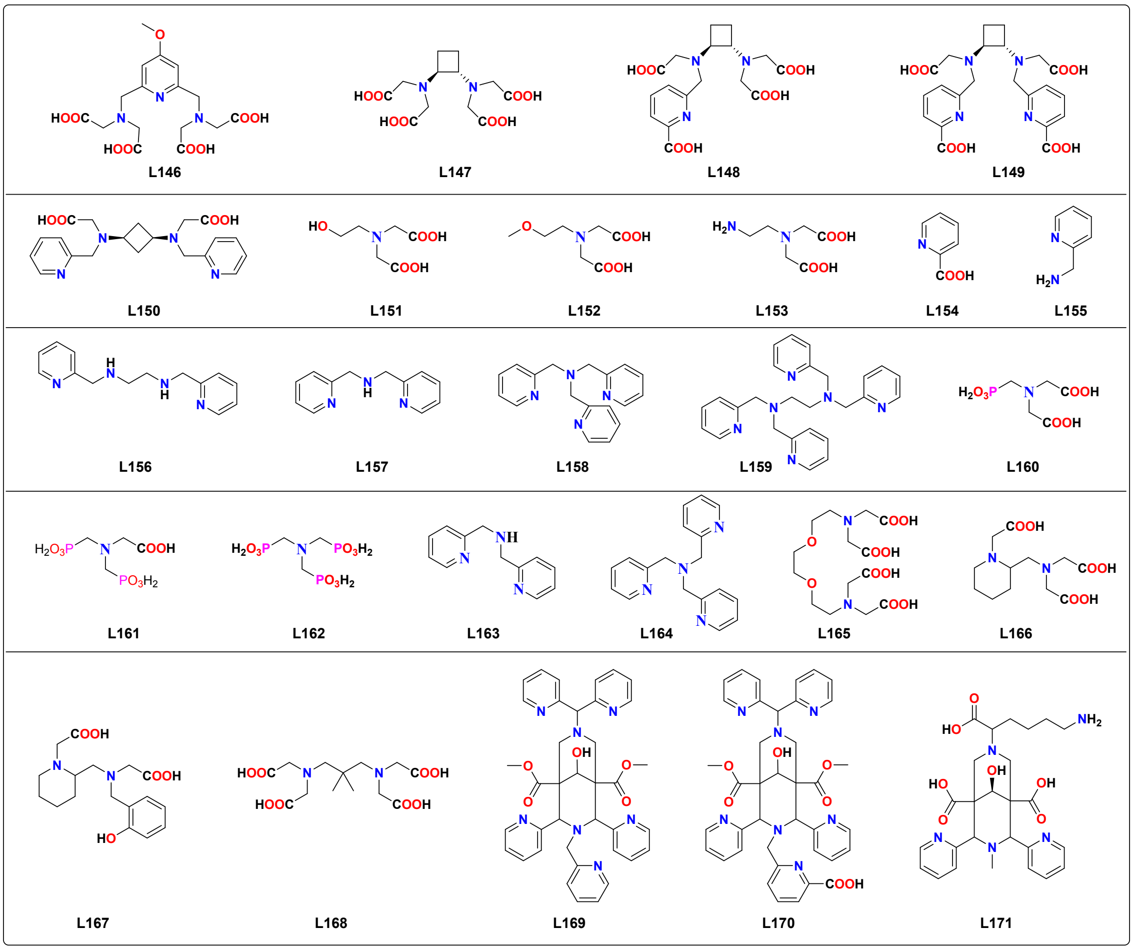

References

(1) Holloway, J. H.; Reilley, C. N. Metal Chelate Stability Constants of Aminopolycarboxylate Ligands. *Anal. Chem.* **1960**, *32* (2), 249–256. https://doi.org/10.1021/ac60158a033.

(2) Negri, R.; Baranyai, Z.; Tei, L.; Giovenzana, G. B.; Platas-Iglesias, C.; Bényei, A. C.; Bodnár, J.; Vágner, A.; Botta, M. Lower Denticity Leading to Higher Stability: Structural and Solution Studies of Ln(III)–OBETA Complexes. *Inorg. Chem.* **2014**, *53* (23), 12499–12511. https://doi.org/10.1021/ic5020225.

(3) Forgács, A.; Giovenzana, G. B.; Botta, M.; Brücher, E.; Tóth, I.; Baranyai, Z. Influence of Gem-Dimethyl Substitution on the Stability, Kinetics and Relaxometric Properties of PDTA Complexes. *Eur. J. Inorg. Chem.* **2012**, *2012* (12), 2074–2086. https://doi.org/10.1002/ejic.201101294.

(4) Kálmán, F. K.; Tircsó, G. Kinetic Inertness of the Mn^{2+} Complexes Formed with AAZTA and Some Open-Chain EDTA Derivatives. *Inorg. Chem.* **2012**, *51* (19), 10065–10067.

(5) Uzal-Varela, R.; Lalli, D.; Brandariz, I.; Rodríguez-Rodríguez, A.; Platas-Iglesias, C.; Botta, M.; Esteban-Gómez, D. Rigid Versions of $PDTA^{4-}$ Incorporating a 1,3-Diaminocyclobutyl Spacer for Mn^{2+} Complexation: Stability, Water Exchange Dynamics and Relaxivity. *Dalton Trans.* **2021**, *50* (44), 16290–16303. https://doi.org/10.1039/D1DT02498A.

(6) Irving, H. M. N. H.; Sharpe, K. Divalent Metal Complexes of Meso- and Dl-2,3-Diaminobutane-N,N,N′,N′-Tetra-Acetic Acid. *J. Inorg. Nucl. Chem.* **1971**, *33* (1), 203–215. https://doi.org/10.1016/0022-1902(71)80022-2.

(7) Sawada, K.; Duan, W.; Ono, M.; Satoh, K. Stability and Structure of Nitrilo(Acetate–Methylphosphonate) Complexes of the Alkaline-Earth and Divalent Transition Metal Ions in Aqueous Solution. *J. Chem. Soc. Dalton Trans.* **2000**, No. 6, 919–924. https://doi.org/10.1039/a909207b.

(8) Laine, S.; Bonnet, C. S.; Kálmán, F. K.; Garda, Z.; Pallier, A.; Caillé, F.; Suzenet, F.; Tircsó, G.; Tóth, É. Mn^{2+} Complexes of Open-Chain Ligands with a Pyridine Backbone: Less Donor Atoms Lead to Higher Kinetic Inertness. *New J. Chem.* **2018**, *42* (10), 8012–8020. https://doi.org/10.1039/C8NJ00648B.

(9) Su, H.; Wu, C.; Zhu, J.; Miao, T.; Wang, D.; Xia, C.; Zhao, X.; Gong, Q.; Song, B.; Ai, H. Rigid Mn(Ii) Chelate as Efficient MRI Contrast Agent for Vascular

Imaging. *Dalton Trans.* **2012**, *41* (48), 14480–14483. https://doi.org/10.1039/c2dt31696j.

(10) Gale, E. M.; Atanasova, I. P.; Blasi, F.; Ay, I.; Caravan, P. A Manganese Alternative to Gadolinium for MRI Contrast. *J. Am. Chem. Soc.* **2015**, *137* (49), 15548–15557.

(11) Rocklage, S. M.; Cacheris, W. P.; Quay, S. C.; Hahn, F. E.; Raymond, K. N. Manganese(II) N,N'-Dipyridoxylethylenediamine-N,N'-Diacetate 5,5'-Bis(Phosphate). Synthesis and Characterization of a Paramagnetic Chelate for Magnetic Resonance Imaging Enhancement. *Inorg. Chem.* **1989**, *28* (3), 477–485.

(12) Uzal-Varela, R.; Rodríguez-Rodríguez, A.; Martínez-Calvo, M.; Carniato, F.; Lalli, D.; Esteban-Gómez, D.; Brandariz, I.; Pérez-Lourido, P.; Botta, M.; Platas-Iglesias, C. Mn^{2+} Complexes Containing Sulfonamide Groups with PH-Responsive Relaxivity. *Inorg. Chem.* **2020**, *59* (19), 14306–14317.

(13) Forgács, A.; Pujales-Paradela, R.; Regueiro-Figueroa, M.; Valencia, L.; Esteban-Gómez, D.; Botta, M.; Platas-Iglesias, C. Developing the Family of Picolinate Ligands for Mn^{2+} Complexation. *Dalton Trans.* **2017**, *46* (5), 1546–1558.

(14) Gale, E. M.; Mukherjee, S.; Liu, C.; Loving, G. S.; Caravan, P. Structure–Redox–Relaxivity Relationships for Redox Responsive Manganese-Based Magnetic Resonance Imaging Probes. *Inorg. Chem.* **2014**, *53* (19), 10748–10761. https://doi.org/10.1021/ic502005u.

(15) Molnár, E.; Váradi, B.; Garda, Z.; Botár, R.; Kálmán, F.; Tóth, É.; Toth, I.; Brücher, E.; Tircsó, G. Remarkable Differences and Similarities between the Isomeric Mn(II)- Cis - and Trans- 1,2-Diaminocyclohexane- N , N , N ', N '-Tetraacetate Complexes. *Inorganica Chim. Acta* **2018**, *472*, 254–263.

(16) Pota, K.; Garda, Z.; Kálmán, F. K.; Barriada, J. L.; Esteban-Gómez, D.; Platas-Iglesias, C.; Tóth, I.; Brücher, E.; Tircsó, G. Taking the next Step toward Inert Mn^{2+} Complexes of Open-Chain Ligands: The Case of the Rigid PhDTA Ligand. *New J. Chem.* **2018**, *42* (10), 8001–8011. https://doi.org/10.1039/C8NJ00121A.

(17) Garda, Z.; Forgács, A.; Do, Q. N.; Kálmán, F. K.; Timári, S.; Baranyai, Z.; Tei, L.; Tóth, I.; Kovács, Z.; Tircsó, G. Physico-Chemical Properties of MnII Complexes Formed with Cis- and Trans-DO2A: Thermodynamic, Electrochemical and Kinetic Studies. *J. Inorg. Biochem.* **2016**, *163*, 206–213. https://doi.org/10.1016/j.jinorgbio.2016.07.018.

(18) Garda, Z.; Molnár, E.; Kálmán, F. K.; Botár, R.; Nagy, V.; Baranyai, Z.; Brücher, E.; Kovács, Z.; Tóth, I.; Tircsó, G. Effect of the Nature of Donor Atoms on the Thermodynamic, Kinetic and Relaxation Properties of Mn(II) Complexes Formed With Some Trisubstituted 12-Membered Macrocyclic Ligands. *Front. Chem.* **2018**, *6* (232).

(19) Kálmán, F. K.; Nagy, V.; Uzal-Varela, R.; Pérez-Lourido, P.; Esteban-Gómez, D.; Garda, Z.; Pota, K.; Mezei, R.; Pallier, A.; Tóth, É.; Platas-Iglesias, C.; Tircsó, G. Expanding the Ligand Classes Used for Mn(II) Complexation: Oxa-Aza Macrocycles Make the Difference. *Molecules* **2021**, *26* (6), 1524. https://doi.org/10.3390/molecules26061524.

(20) Chaves, S.; Delgado, R.; Da Silva, J. J. R. F. The Stability of the Metal Complexes of Cyclic Tetra-Aza Tetra-Acetic Acids. *Talanta* **1992**, *39* (3), 249–254. https://doi.org/10.1016/0039-9140(92)80028-C.

(21) Takács, A.; Napolitano, R.; Purgel, M.; Bényei, A. C.; Zékány, L.; Brücher, E.; Tóth, I.; Baranyai, Z.; Aime, S. Solution Structures, Stabilities, Kinetics, and Dynamics of DO3A and DO3A–Sulphonamide Complexes. *Inorg. Chem.* **2014**, *53* (6), 2858–2872. https://doi.org/10.1021/ic4025958.

(22) Forgács, A.; Tei, L.; Baranyai, Z.; Esteban-Gómez, D.; Platas-Iglesias, C.; Botta, M. Optimising the Relaxivities of Mn^{2+} Complexes by Targeting Human Serum Albumin (HSA). *Dalton Trans.* **2017**, *46* (26), 8494–8504. https://doi.org/10.1039/C7DT01508A.

(23) Bárta, J.; Hermann, P.; Kotek, J. Coordination Behavior of 1,4-Disubstituted Cyclen Endowed with Phosphonate, Phosphonate Monoethylester, and H-Phosphinate Pendant Arms. *Molecules* **2019**, *24* (18), 3324. https://doi.org/10.3390/molecules24183324.

(24) Csupász, T.; Szücs, D.; Kálmán, F. K.; Hollóczki, O.; Fekete, A.; Szikra, D.; Tóth, É.; Tóth, I.; Tircsó, G. A New Oxygen Containing Pyclen-Type Ligand as a Manganese(II) Binder for MRI and 52Mn PET Applications: Equilibrium, Kinetic, Relaxometric, Structural and Radiochemical Studies. *Molecules* **2022**, *27* (2), 371. https://doi.org/10.3390/molecules27020371.

(25) Forgács, A.; Tei, L.; Baranyai, Z.; Tóth, I.; Zékány, L.; Botta, M. A Bisamide Derivative of [Mn(1,4-DO2A)] - Solution Thermodynamic, Kinetic, and NMR Relaxometric Studies. *Eur. J. Inorg. Chem.* **2016**, *2016* (8), 1165–1174. https://doi.org/10.1002/ejic.201501415.

(26) Garda, Z.; Molnár, E.; Hamon, N.; Barriada, J. L.; Esteban-Gómez, D.; Váradi, B.; Nagy, V.; Pota, K.; Kálmán, F. K.; Tóth, I.; Lihi, N.; Platas-Iglesias, C.; Tóth, É.; Tripier, R.; Tircsó, G. Complexation of Mn(II) by Rigid Pyclen Diacetates: Equilibrium, Kinetic, Relaxometric, Density Functional Theory, and Superoxide Dismutase Activity Studies. *Inorg. Chem.* **2021**, *60* (2), 1133–1148. https://doi.org/10.1021/acs.inorgchem.0c03276.

(27) Drahoš, B.; Pniok, M.; Havlíčková, J.; Kotek, J.; Císařová, I.; Hermann, P.; Lukeš, I.; Tóth, É. Mn2+ Complexes of 1-Oxa-4,7-Diazacyclononane Based Ligands with Acetic, Phosphonic and Phosphinic Acid Pendant Arms: Stability and Relaxation Studies. *Dalton Trans.* **2011**, *40* (39), 10131–10146. https://doi.org/10.1039/c1dt10543d.

(28) Lázár, I.; Király, R.; Takács, Z. Synthesis, Potentiometric and ^{1}H NMR Study of Protonation and Complex Formation of 1,4,7-Triazacyclononane-1,4-Diacetate. *J. Coord. Chem.* **2000**, *51* (2), 293–304. https://doi.org/10.1080/00958970008055135.

(29) Cabral, M. F.; Costa, J.; Delgado, R.; Da Silva, J. J. R. F.; Vilhena, M. F. Protonation and Metal Complexation Studies on Some Oxa-Diaza Macrocyclic Ligands. *Polyhedron* **1990**, *9* (23), 2847–2857. https://doi.org/10.1016/S0277-5387(00)84189-3.

(30) Tei, L.; Gugliotta, G.; Fekete, M.; Kálmán, F. K.; Botta, M. Mn(II) Complexes of Novel Hexadentate AAZTA-like Chelators: A Solution Thermodynamics and Relaxometric Study. *Dalton Trans.* **2011**, *40* (9), 2025–2032. https://doi.org/10.1039/c0dt01114b.

(31) Riley, D. P.; Henke, S. L.; Lennon, P. J.; Weiss, R. H.; Neumann, W. L.; Rivers, W. J.; Aston, K. W.; Sample, K. R.; Rahman, H.; Ling, C.-S.; Shieh, J.-J.; Busch, D. H.; Witold Szulbinski. Synthesis, Characterization, and Stability of Manganese(II) C-Substituted 1,4,7,10,13-Pentaazacyclopentadecane Complexes Exhibiting Superoxide Dismutase Activity. *Inorg. Chem.* **1996**, *35* (18), 5213–5231. https://doi.org/10.1021/ic960262v.

(32) Drahoš, B.; Kotek, J.; Hermann, P.; Lukeš, I.; Tóth, É. Mn $^{2+}$ Complexes with Pyridine-Containing 15-Membered Macrocycles: Thermodynamic, Kinetic, Crystallographic, and ^{1} H/ ^{17} O Relaxation Studies. *Inorg. Chem.* **2010**, *49* (7), 3224–3238.

(33) Costa, J.; Delgado, R. Metal Complexes of Macrocyclic Ligands Containing Pyridine. *Inorg. Chem.* **1993**, *32* (23), 5257–5265. https://doi.org/10.1021/ic00075a052.

(34) Bianchi, A.; Calabi, L.; Giorgi, C.; Losi, P.; Mariani, P.; Palano, D.; Paoli, P.; Rossi, P.; Valtancoli, B. Thermodynamic and Structural Aspects of Manganese(II) Complexes with Polyaminopolycarboxylic Ligands Based upon 1,4,7,10-Tetraazacyclododecane (Cyclen). Crystal Structure of Dimeric [MnL]2·2CH3OH Containing the New Ligand 1,4,7,10-Tetraazacyclododecane-1,4-Diacetate. *J. Chem. Soc. Dalton Trans.* **2001**, No. 6, 917–922. https://doi.org/10.1039/B009242H.

(35) Bianchi, A.; Calabi, L.; Giorgi, C.; Losi, P.; Mariani, P.; Paoli, P.; Rossi, P.; Valtancoli, B.; Virtuani, M. Thermodynamic and Structural Properties of Gd^{3+} Complexes with Functionalized Macrocyclic Ligands Based upon 1,4,7,10-Tetraazacyclododecane. *J. Chem. Soc. Dalton Trans.* **2000**, No. 5, 697–705. https://doi.org/10.1039/A909098C.

(36) Aime, S.; Anelli, P.; Botta, M.; Brocchetta, M.; Canton, S.; Fedeli, F.; Gianolio, E.; Terreno, E. Relaxometric Evaluation of Novel Manganese(II) Complexes for Application as Contrast Agents in Magnetic Resonance Imaging. *JBIC J. Biol. Inorg. Chem.* **2002**, *7* (1–2), 58–67. https://doi.org/10.1007/s007750100265.

(37) Botár, R.; Molnár, E.; Trencsényi, G.; Kiss, J.; Kálmán, F. K.; Tircsó, G. Stable and Inert Mn(II)-Based and pH-Responsive Contrast Agents. *J. Am. Chem. Soc.* **2020**, *142* (4), 1662–1666.

(38) Drahoš, B.; Kotek, J.; Císařová, I.; Hermann, P.; Helm, L.; Lukeš, I.; Tóth, É. Mn^{2+} Complexes with 12-Membered Pyridine Based Macrocycles Bearing Carboxylate or Phosphonate Pendant Arm: Crystallographic, Thermodynamic, Kinetic, Redox, and ^{1}H/ ^{17}O Relaxation Studies. *Inorg. Chem.* **2011**, *50* (24), 12785–12801.

(39) Delgado, R.; Quintino, S.; Teixeira, M.; Zhang, A. Metal Complexes of a 12-Membered Tetraaza Macrocycle Containingpyridine and N-Carboxymethyl Groups. *J. Chem. Soc. Dalton Trans.* **1997**, No. 1, 55–64. https://doi.org/10.1039/A602311H.

(40) Failli, P.; Bani, D.; Bencini, A.; Cantore, M.; Di Cesare Mannelli, L.; Ghelardini, C.; Giorgi, C.; Innocenti, M.; Rugi, F.; Spepi, A.; Udisti, R.; Valtancoli, B. A Novel Manganese Complex Effective as Superoxide Anion Scavenger and

Therapeutic Agent against Cell and Tissue Oxidative Injury. *J. Med. Chem.* **2009**, *52* (22), 7273–7283. https://doi.org/10.1021/jm901298x.

(41) Botár, R.; Molnár, E.; Garda, Z.; Madarasi, E.; Trencsényi, G.; Kiss, J.; Kálmán, F. K.; Tircsó, G. Synthesis and Characterization of a Stable and Inert Mn^{II} -Based Zn^{II} Responsive MRI Probe for Molecular Imaging of Glucose Stimulated Zinc Secretion (GSZS). *Inorg. Chem. Front.* **2022**, *9* (3), 577–583. https://doi.org/10.1039/D1QI00501D.

(42) Kálmán, F. K.; Nagy, V.; Váradi, B.; Garda, Z.; Molnár, E.; Trencsényi, G.; Kiss, J.; Même, S.; Même, W.; Tóth, É.; Tircsó, G. Mn(II)-Based MRI Contrast Agent Candidate for Vascular Imaging. *J. Med. Chem.* **2020**, *63* (11), 6057–6065. https://doi.org/10.1021/acs.jmedchem.0c00197.

(43) Svobodová, I.; Lubal, P.; Plutnar, J.; Havlíčková, J.; Kotek, J.; Hermann, P.; Lukeš, I. Thermodynamic, Kinetic and Solid-State Study of Divalent Metal Complexes of 1,4,8,11-Tetraazacyclotetradecane (Cyclam) Bearing Two Trans (1,8-)Methylphosphonic Acid Pendant Arms. *Dalton Trans* **2006**, No. 43, 5184–5197. https://doi.org/10.1039/B603251F.

(44) Molnár, E.; Camus, N.; Patinec, V.; Rolla, G. A.; Botta, M.; Tircsó, G.; Kálmán, F. K.; Fodor, T.; Tripier, R.; Platas-Iglesias, C. Picolinate-Containing Macrocyclic Mn^{2+} Complexes as Potential MRI Contrast Agents. *Inorg. Chem.* **2014**, *53* (10), 5136–5149. https://doi.org/10.1021/ic500231z.

(45) Amorim, M. T. S.; Delgado, R.; da Silva, J. J. R. F. N,N′-Diacetate Derivatives of Some Polyoxa-Polyaza Macrocyclic Compounds: Protonation and Complexation Studies. *Polyhedron* **1992**, *11* (15), 1891–1899. https://doi.org/10.1016/S0277-5387(00)83737-7.

(46) Costa, J.; Delgado, R.; Drew, M. G. B.; Félix, V. Design of Selective Macrocyclic Ligands for the Divalent First-Row Transition-Metal Ions. *J. Chem. Soc. Dalton Trans.* **1998**, No. 6, 1063–1072. https://doi.org/10.1039/a706434i.

(47) Dees, A.; Zahl, A.; Puchta, R.; van Eikema Hommes, N. J. R.; Heinemann, F. W.; Ivanović-Burmazović, I. Water Exchange on Seven-Coordinate Mn(II) Complexes with Macrocyclic Pentadentate Ligands: Insight in the Mechanism of Mn(II) SOD Mimetics. *Inorg. Chem.* **2007**, *46* (7), 2459–2470. https://doi.org/10.1021/ic061852o.

(48) Lieb, D.; Friedel, F. C.; Yawer, M.; Zahl, A.; Khusniyarov, M. M.; Heinemann, F. W.; Ivanović-Burmazović, I. Dinuclear Seven-Coordinate Mn(II) Complexes:

Effect of Manganese(II)-Hydroxo Species on Water Exchange and Superoxide Dismutase Activity. *Inorg. Chem.* **2013**, *52* (1), 222–236. https://doi.org/10.1021/ic301714d.

(49) Pota, K.; Molnár, E.; Kálmán, F. K.; Freire, D. M.; Tircsó, G.; Green, K. N. Manganese Complex of a Rigidified 15-Membered Macrocycle: A Comprehensive Study. *Inorg. Chem.* **2020**, *59* (16), 11366–11376. https://doi.org/10.1021/acs.inorgchem.0c01053.

(50) Cabral, M. F.; Delgado, R. 4,7,10,13-Tetrakis(Carboxymethyl)-1-Oxa-4,7,10,13-Tetraazacyclopentadecane and Properties of Its Metal Complexes. *Polyhedron* **1999**, *18* (26), 3479–3489. https://doi.org/10.1016/S0277-5387(99)00258-2.

(51) Yang, Rita.; Zompa, L. J. Metal Complexes of Cyclic Triamines. 1. Complexes of 1,4,7-Triazacyclononane ([9]AneN3) with Nickel(II), Copper(II), and Zinc(II). *Inorg. Chem.* **1976**, *15* (7), 1499–1502. https://doi.org/10.1021/ic50161a007.

(52) de Sá, A.; Bonnet, C. S.; Geraldes, C. F. G. C.; Tóth, É.; Ferreira, P. M. T.; André, J. P. Thermodynamic Stability and Relaxation Studies of Small, Triaza-Macrocyclic Mn(Ii) Chelates. *Dalton Trans.* **2013**, *42* (13), 4522–4532. https://doi.org/10.1039/c2dt32496b.

(53) Van der Merwe, M. J.; Boeyens, J. C. A.; Hancock, R. D. Crystallographic and Thermodynamic Study of Metal Ion Size Selectivity in the Ligand 1,4,7-Triazacyclononane-N,N',N"-Triacetate. *Inorg. Chem.* **1985**, *24* (8), 1208–1213. https://doi.org/10.1021/ic00202a019.

(54) Cortes, S.; Brucher, E.; Geraldes, C. F. G. C.; Sherry, A. D. Potentiometry and NMR Studies of 1,5,9-Triazacyclododecane-N,N',N''-Triacetic Acid and Its Metal Ion Complexes. *Inorg. Chem.* **1990**, *29* (1), 5–9. https://doi.org/10.1021/ic00326a003.

(55) Kabachnik, M. I.; Medved', T. Y.; Polikarpov, Y. M.; Shcherbakov, B. K.; Bel'skii, F. I.; Pasechnik, M. P. Synthesis and Study of a New Complexone-N,N′,N″-Tris-(Dihydroxyphosphorylmethyl)-1,4,7-Triazacyclononane. *Bull. Acad. Sci. USSR Div. Chem. Sci.* **1984**, *33*, 769–777.

(56) Guillou, A.; Galland, M.; Roux, A.; Váradi, B.; Gogolák, R. A.; Le Saëc, P.; Faivre-Chauvet, A.; Beyler, M.; Bucher, C.; Tircsó, G.; Patinec, V.; Maury, O.; Tripier, R. Picolinate-Appended Tacn Complexes for Bimodal Imaging: Radiolabeling, Relaxivity, Photophysical and Electrochemical Studies. *J. Inorg. Biochem.* **2020**, *205*, 110978. https://doi.org/10.1016/j.jinorgbio.2019.110978.

(57) Holyer, R. H.; Hubbard, C. D.; Kettle, S. F. A.; Wilkins, R. G. The Kinetics of Replacement Reactions of Complexes of the Transition Metals with 2,2',2"-Terpyridine. *Inorg. Chem.* **1966**, *5* (4), 622–625. https://doi.org/10.1021/ic50038a027.

(58) Vanasschen, C.; Molnár, E.; Tircsó, G.; Kálmán, F. K.; Tóth, É.; Brandt, M.; Coenen, H. H.; Neumaier, B. Novel CDTA-Based, Bifunctional Chelators for Stable and Inert Mn^{II} Complexation: Synthesis and Physicochemical Characterization. *Inorg. Chem.* **2017**, *56* (14), 7746–7760.

(59) Anderegg, G.; Nägeli, P.; Müller, F.; Schwarzenbach, G. Komplexone XXX. Diäthylentriamin-pentaessigsäure (DTPA). *Helv. Chim. Acta* **1959**, *42* (3), 827–836. https://doi.org/10.1002/hlca.19590420324.

(60) Anderegg, G. Komplexone XL. Die Protonierungskonstanten einiger Komplexone in verschiedenen wässerigen Salzmedien ($NaClO_4$, $(CH_3)_4$ NCl, KNO_3). *Helv. Chim. Acta* **1967**, *50* (8), 2333–2340. https://doi.org/10.1002/hlca.19670500818.

(61) Ogino, H. The Stability Constants of Ethylenediaminetetraacetato, Trimethylenediaminetetraacetato and Propylenediaminetetraacetato Complexes of Some Divalent Metal Ions. *Bull. Chem. Soc. Jpn.* **1965**, *38* (5), 771–777.

(62) Nakasuka, N.; Kunimatsu, M.; Matsumura, K.; Tanaka, M. Complexation of O-Phenylenediaminetetraacetic Acid with Some Bivalent First-Row Transition-Metal Ions in Aqueous Solution. *Inorg. Chem.* **1985**, *24* (1), 10–15. https://doi.org/10.1021/ic00195a005.

(63) Forgács, A.; Regueiro-Figueroa, M.; Barriada, J. L.; Esteban-Gómez, D.; de Blas, A.; Rodríguez-Blas, T.; Botta, M.; Platas-Iglesias, C. Mono-, Bi-, and Trinuclear Bis-Hydrated Mn^{2+} Complexes as Potential MRI Contrast Agents. *Inorg. Chem.* **2015**, *54* (19), 9576–9587.

(64) Leporati, E. Potentiometric Study of the Complex-Formation Equilibria of Manganese(II), Cobalt(II), Nickel(II), Copper(II), and Zinc(II) with Ethylenediamine-N-Acetic Acid. *J. Chem. Soc. Dalton Trans.* **1985**, No. 8, 1605. https://doi.org/10.1039/dt9850001605.

(65) Hancock, R. D. The Chelate Effect in Complexes with Ethanolamine. *Inorganica Chim. Acta* **1981**, *49*, 145–148. https://doi.org/10.1016/S0020-1693(00)90474-2.

(66) Eplattenier, F. L.; Murase, I.; Martell, A. E. New Multidentate Ligands. VI. Chelating Tendencies of N,N′-Di(2-Hydroxybenzyl)Ethylenediamine-N,N′-

Diacetic Acid. *J. Am. Chem. Soc.* **1967**, *89* (4), 837–843. https://doi.org/10.1021/ja00980a019.

(67) Chaberek, S.; Martell, A. E. Interaction of Divalent Metal Ions with N-Hydroxyethylethylenediaminetriacetic Acid. *J. Am. Chem. Soc.* **1955**, *77* (6), 1477–1480. https://doi.org/10.1021/ja01611a022.

(68) Motekaitis, R. J.; Martell, A. E. METAL CHELATE FORMATION BY *N* - PHOSPHONOMETHYLGLYCINE AND RELATED LIGANDS. *J. Coord. Chem.* **1985**, *14* (2), 139–149. https://doi.org/10.1080/00958978508073900.

(69) Baseggio, A. A.; Grassi, R. L. Stability Constants of the Complexes of Zn(II) and Mn(II) with N,N′-Bis(2-Hydroxyethyl) Aminomethyl. *J. Inorg. Nucl. Chem.* **1981**, *43* (12), 3275–3276. https://doi.org/10.1016/0022-1902(81)80101-7.

(70) Pujales-Paradela, R.; Carniato, F.; Uzal-Varela, R.; Brandariz, I.; Iglesias, E.; Platas-Iglesias, C.; Botta, M.; Esteban-Gómez, D. A Pentadentate Member of the Picolinate Family for Mn(II) Complexation and an Amphiphilic Derivative. *Dalton Trans.* **2019**, *48* (2), 696–710. https://doi.org/10.1039/C8DT03856B.

(71) Paoletti, P.; Vacca, A. Thermochemical Studies. Payt XIII. Heats and Entropies of Reaction of Tetraethylenepentamine with Protons and Bivalent Transition-Metal Ions. *J. Chem. Soc.* **1964**, 5051–5057.

(72) Lacoste, R. G.; Kartell, A. E. New Multidentate Ligands. I. Coordinating Tendencies of Polyamines Containing -Pyridyl Groups with Divalent Metal Ions12. *Inorg. Chem.* **1964**, *3* (6), 881–884.

(73) Anderegg, G. PYRIDINE DERIVATIVES AS COMPLEXING AGENTS XII. Thermodynamics of Complex Formation with 2-Pyridylmethyl-Iminodiacetic Acid and Its 6-Methyl Substituted Derivative. *J. Coord. Chem.* **1981**, *11* (3), 171–175. https://doi.org/10.1080/00958978108079065.

(74) Rocklage, S. M.; Sheffer, S. H.; Cacheris, W. P.; Quay, S. C.; Hahn, E. F.; Raymond, K. N. Structural and Thermodynamic Characterization of Manganese(II) N,N'-Dipyridoxylethylenediamine-N,N'-Diacetate. A Novel Manganese(II) Chelate. *Inorg. Chem.* **1988**, *27* (20), 3530–3534. https://doi.org/10.1021/ic00293a020.

(75) Porcar-Tost, O.; Pallier, A.; Esteban-Gómez, D.; Illa, O.; Platas-Iglesias, C.; Tóth, É.; Ortuño, R. M. Stability, Relaxometric and Computational Studies on Mn^{2+} Complexes with Ligands Containing a Cyclobutane Scaffold. *Dalton Trans.* **2021**, *50* (3), 1076–1085. https://doi.org/10.1039/D0DT03402A.

(76) Chaberek, S.; Courtney, R. C.; Martell, A. E. Stability of Metal Chelates. II. /3-Hy Droxy Ethyliminodiacetic Acid. *J. Am. Chem. Soc.* **1952**, *74*, 5057–5060.
(77) Schwarzenbach, G.; Anderegg, G.; Schneider, W.; Senn, H. Komplexone XXVI. Über die Koordinationstendenz von N-substituierten Iminodiessigsäuren. *Helvetia Chim. Acta* **1955**, *38*, 1147–1170.
(78) Anderegg, G. Pyridinderivate als Komplexbildner I. Pyridincarbonsäuren. *Helv. Chim. Acta* **1960**, *43* (1), 414–424. https://doi.org/10.1002/hlca.19600430153.
(79) Anderegg, G. Pyridinderivate als Komplexbildner IX Die Stabilitätskonstanten von Komplexen mit (a) 2-aminomethyl-pyridin, (b) 6-methyl-2-aminomethyl-pyridin, (c) 2-pyridylhydrazin, (d) 2,2'-dipyridylamin und (e) 1-(?-pyridylmethylen)-2-(?'-pyridyl)-hydrazin. *Helv. Chim. Acta* **1971**, *54* (2), 509–512. https://doi.org/10.1002/hlca.19710540209.
(80) Anderegg, G.; Wenk, F. Pyridinderivate als Komplexbildner VIII Die Herstellung je eines neuen vier- und sechszähnigen Liganden. *Helv. Chim. Acta* **1967**, *50* (8), 2330–2332. https://doi.org/10.1002/hlca.19670500817.
(81) Kripli, B.; Garda, Z.; Sólyom, B.; Tircsó, G.; Kaizer, J. Formation, Stability and Catalase-like Activity of Mononuclear Manganese(II) and Oxomanganese(IV) Complexes in Protic and Aprotic Solvents. *New J. Chem.* **2020**, *44* (14), 5545–5555. https://doi.org/10.1039/C9NJ06004A.
(82) Martinelli, J.; Callegari, E.; Baranyai, Z.; Fraccarollo, A.; Cossi, M.; Tei, L. Semi-Rigid (Aminomethyl) Piperidine-Based Pentadentate Ligands for Mn(II) Complexation. *Molecules* **2021**, *26* (19), 5993. https://doi.org/10.3390/molecules26195993.
(83) Cieslik, P.; Comba, P.; Dittmar, B.; Ndiaye, D.; Tóth, É.; Velmurugan, G.; Wadepohl, H. Exceptional Manganese(II) Stability and Manganese(II)/Zinc(II) Selectivity with Rigid Polydentate Ligands**. *Angew. Chem. Int. Ed.* **2022**, *61* (10), e202115580. https://doi.org/10.1002/anie.202115580.
(84) Ndiaye, D.; Sy, M.; Pallier, A.; Même, S.; Silva, I.; Lacerda, S.; Nonat, A. M.; Charbonnière, L. J.; Tóth, É. Unprecedented Kinetic Inertness for a Mn^{2+} -Bispidine Chelate: A Novel Structural Entry for Mn^{2+} -Based Imaging Agents. *Angew. Chem. Int. Ed.* **2020**, *59* (29), 11958–11963.
(85) APEX3 Version 2016.1; Bruker AXS Inc., 2016.
(86) SAINT Version 8.38A; Bruker AXS Inc., 2015.
(87) Sheldrick, G. M.SADABS Version 2014/5; Bruker AXS Inc.

(88) Sheldrick, G. M. Crystal Structure Refinement with SHELXL Version 2014/5. *Acta Cryst. Sect C Struct. Chem.* **2015**, *71* (1), 3–8.

(89) Sheldrick, G. M. A Short History of SHELX. *Acta Crystallogr. A* **2008**, *64* (1), 112-122 (SHELXL Version 2014/7).

(90) Dolomanov, O. V.; Bourhis, L. J.; Gildea, R. J.; Howard, J. A. K.; Puschmann, H. OLEX2: A Complete Structure Solution, Refinement and Analysis Program. *J. Appl. Crystallogr.,* **2009**, *42*, 339–341.
